# Bidirectional multiciliated cell extrusion is controlled by Notch-driven basal extrusion and Piezo1-driven apical extrusion

**DOI:** 10.1242/dev.201612

**Published:** 2023-09-01

**Authors:** Rosa Ventrella, Sun K. Kim, Jennifer Sheridan, Aline Grata, Enzo Bresteau, Osama A. Hassan, Eve E. Suva, Peter Walentek, Brian J. Mitchell

**Affiliations:** ^1^ Northwestern University, Feinberg School of Medicine, Department of Cell and Developmental Biology, Chicago, IL 60611, USA; ^2^ Precision Medicine Program, Midwestern University, Downers Grove, IL 60515, USA; ^3^ University of Freiburg, Renal Division, Internal Medicine IV, Medical Center and CIBSS Centre for Integrative Biological Signalling Studies, 79104 Freiburg im Breisgau, Germany; ^4^ Northwestern University, Lurie Cancer Center, Chicago, IL 60611, USA

**Keywords:** Multiciliated cells, Cell extrusion, Notch, Piezo1

## Abstract

*Xenopus* embryos are covered with a complex epithelium containing numerous multiciliated cells (MCCs). During late-stage development, there is a dramatic remodeling of the epithelium that involves the complete loss of MCCs. Cell extrusion is a well-characterized process for driving cell loss while maintaining epithelial barrier function. Normal cell extrusion is typically unidirectional, whereas bidirectional extrusion is often associated with disease (e.g. cancer). We describe two distinct mechanisms for MCC extrusion, a basal extrusion driven by Notch signaling and an apical extrusion driven by Piezo1. Early in the process there is a strong bias towards basal extrusion, but as development continues there is a shift towards apical extrusion. Importantly, response to the Notch signal is age dependent and governed by the maintenance of the MCC transcriptional program such that extension of this program is protective against cell loss. In contrast, later apical extrusion is regulated by Piezo1, such that premature activation of Piezo1 leads to early extrusion while blocking Piezo1 leads to MCC maintenance. Distinct mechanisms for MCC loss underlie the importance of their removal during epithelial remodeling.

## Teaser

**Summary:** Notch-driven basal extrusion and Piezo1-mediated apical extrusion work in concert to ensure that all multiciliated cells are lost from the epithelium by stage 48 of *Xenopus* embryonic development.

## INTRODUCTION

Ciliated epithelial-driven fluid flow is essential for moving mucus and particles across the lungs and oviduct, as well as for cerebrospinal fluid circulation. It is also required for the locomotion of some aquatic animals. In many of these biological contexts, a ciliated epithelium must maintain homeostasis and undergo epithelial remodeling involving the loss and replenishing of multiciliated cells (MCCs) (Rock et al., 2011; Walentek, 2022). *Xenopus* embryos contain a ciliated epithelium comprising numerous MCCs that promote locomotion, protection from infection and gas exchange before lung development. During their development, the need for cilia-driven flow is transient and is ultimately replaced by the swimming motion of the more mature tadpoles. This transition requires a dramatic remodeling of the epithelium that involves the complete loss of MCCs, while maintaining epithelial barrier function.

Cell extrusion is a well-characterized process in the maintenance of epithelial homeostasis and occurs during the remodeling of numerous tissues (Andrade and Rosenblatt, 2011; Rosenblatt et al., 2001; Fadul and Rosenblatt, 2018; Gudipaty et al., 2018; Ohsawa et al., 2018). Cell extrusion from an epithelium takes place in multiple forms, including the loss of unwanted apoptotic cells, the loss of less competitive cells, and the stochastic loss of cells due to overcrowding (Atieh et al., 2021; Eisenhoffer et al., 2012; Ellis et al., 2019; Fadul et al., 2021; Gu et al., 2011; Levayer et al., 2016; Slattum et al., 2014). This removal of cells can occur either basally or apically, depending on the context (Nanavati et al., 2020). Although there is considerable variation across model systems and organs, it is common that, for a particular tissue, cell extrusion typically occurs either apically or basally, but rarely in both directions. Importantly, the incorrect orientation of cell extrusion is often associated with disease states and is well characterized in cancer and hyperplasia (Gu et al., 2015; Gudipaty and Rosenblatt, 2017). Previously, it has been shown that sphingosine 1-phosphate (S1P) and Rho-mediated signaling are required for cell extrusion of both apoptotic and non-apoptotic cells (Eisenhoffer et al., 2012; Gu et al., 2011; Gudipaty and Rosenblatt, 2017). Additionally, the stretch-activated ion channel, Piezo1, has been shown to act as a mechanosensor during epithelial tissue remodeling (Peyronnet et al., 2013; Miyamoto et al., 2014; Gudipaty et al., 2017; Stewart and Davis, 2019) and has been identified as an upstream factor that can drive live cell extrusion (Eisenhoffer et al., 2012).

The transcriptional regulators of the MCC lineage are well characterized (Collins et al., 2021b; Vladar and Mitchell, 2016; Ma et al., 2014; Terre et al., 2016; Stubbs et al., 2012; Boon et al., 2014). In particular, members of the Geminin family of proteins, GemC1 and MCIDAS, form complexes with DP1 and E2F4/5 to promote the transcriptional regulation of the MCC fate (Ma et al., 2014; Terre et al., 2016). In fact, in the proper context, GemC1 and MCIDAS are sufficient and necessary to drive the formation of MCCs (Ma et al., 2014; Terre et al., 2016; Stubbs et al., 2012; Boon et al., 2014; Kim et al., 2018). Interestingly, both GemC1 and MCIDAS are downregulated after the MCC fate is established, whereas downstream transcription factors that drive and maintain cilia formation and function, such as RFX2 and FoxJ1, continue to be expressed throughout the life of the MCC (Briggs et al., 2018; Chung et al., 2014; Chung et al., 2012; Quigley and Kintner, 2017; Stubbs et al., 2012).

Whereas GemC1 and MCIDAS promote MCC specification, Notch signaling acts as a negative regulator of MCC formation. In mammalian lungs and *Xenopus* embryonic skin, activation of Notch signaling inhibits MCC formation, whereas inhibition of Notch results in increased MCC differentiation (Deblandre et al., 1999; Lewis and Stracker, 2021; Liu et al., 2007; Rock et al., 2011). Additionally, the loss of MCCs from the older *Xenopus* epithelium has been shown to require Notch signaling that has been proposed to emanate from the developing lateral line primordium and the underlying mesoderm, based on the expression of Notch ligands (Tasca et al., 2021). Overexpression of the active Notch intracellular domain (NICD) in MCCs has been shown to increase the number of TUNEL staining-positive cells, indicating that Notch can induce apoptosis of MCCs, leading to their loss. Furthermore, the Notch signaling pathway in mouse has been proposed to regulate the transdifferentiation of club cells into MCCs in adult tissue homeostasis when Notch signaling has been blocked (Lafkas et al., 2015).

In this study, we show two distinct mechanisms of MCC extrusion during *Xenopus* embryonic skin remodeling: (1) a Notch-based basal extrusion that requires a cell-autonomous licensing event tied to the loss of MCC transcriptional commitment; and (2) apical extrusion that is regulated by the mechanosensory channel Piezo1. These distinct mechanisms work in concert to ensure that all MCCs are lost from the epithelium by stage (ST) 48 of *Xenopus* embryonic development.

## RESULTS

### Loss of fluid low and multiciliated cells by ST48

**Fig. 1.**
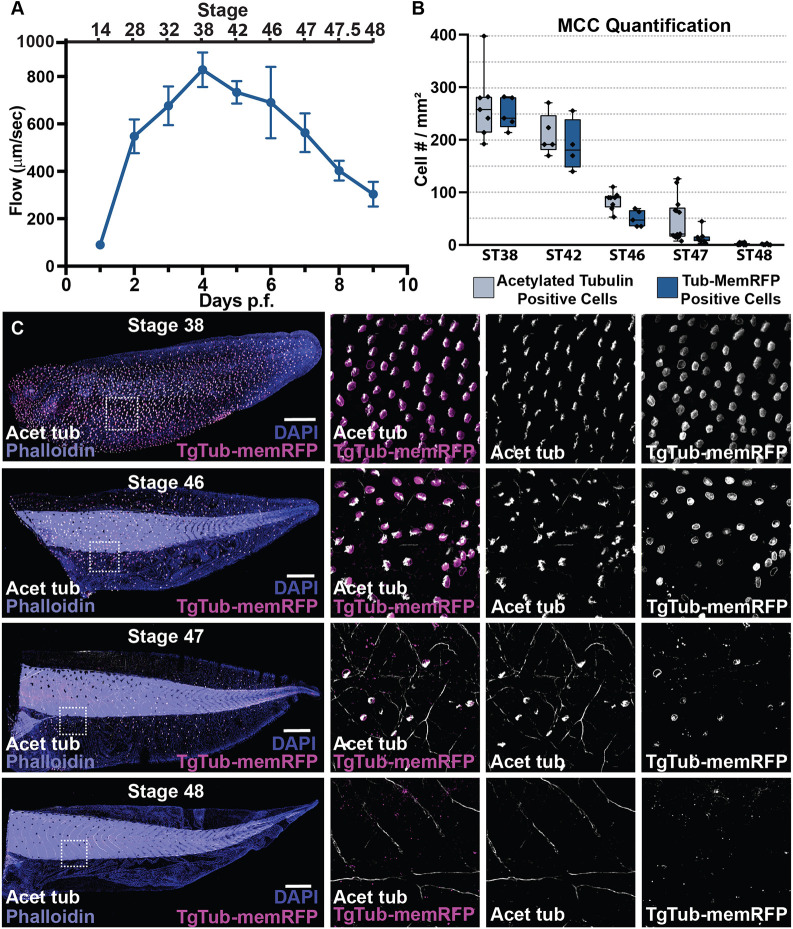
**The loss of cilia-driven fluid flow accompanies the loss of multiciliated cells.** (A) Quantification of cilia-driven fluid flow, as measured by fluorescent bead displacement across the surface of the epithelium showing that flow peaks at 4 dpf and is essentially lost by 9 dpf, *n*=8 animals per time point (data are mean±s.d.). (B) Quantification of MCC number using both the transgenic line TgTub-memRFP driving MCC-specific expression of membrane RFP and antibody staining of acetylated tubulin, *n*>3 embryos per time point. Box and whiskers plot represents 25th-75th percentiles (boxes), minimum and maximum values (whiskers), with the line representing the median. (C) Representative images of the progression of MCC loss in embryos between ST38 and ST48, showing acetylated Tub (white), TgTub-memRFP (magenta), phalloidin (purple) and DAPI (blue). Scale bars: 500 µm.

MCCs work together to drive a directed fluid flow across the surface of the epithelium during *Xenopus* embryonic development. To examine how long MCC-driven fluid flow is maintained, we measured the displacement of fluorescent beads across the embryo's surface (Mitchell et al., 2007; Werner and Mitchell, 2013). We performed a developmental time course of this rate and found that the flow rate peaks 4 days post fertilization (dpf) at ST38. It then steadily decreases over the next few days and is essentially absent by 9 dpf at ST48 (Fig. 1A). This loss of flow corresponds with the progressive loss of cilia observed with acetylated tubulin antibody staining between ST38 and ST48, as previously reported (Fig. 1B,C) (Tasca et al., 2021). Importantly, when using a transgenic line of *Xenopus* that expresses membrane-bound RFP (MyrPalm-mRFP1) specifically in MCCs driven by the α-tubulin A1A (Tub) promoter [Xla.Tg(tuba1a:MyrPalm-mRFP1)NXR; referred to here as TgTub-memRFP], we see a similar time frame of MCC loss (Fig. 1B,C) (Collins et al., 2021a). Importantly, the membrane RFP is stably maintained, and we can visualize cells that have undergone basal cell extrusion and apoptosis (e.g. Fig. 3A,C). Additionally, at later stages of development, we occasionally see some RFP-positive cells devoid of acetylated tubulin, which is consistent with the previous claim that some MCCs are undergoing a trans- or de-differentiation event (Tasca et al., 2021). However, the complete loss of RFP-positive cells by ST48 coupled with the prolonged presence of stable mem-RFP positive cell debris found in the epithelium suggests that this change of fate is transient and that ultimately all of the MCCs are lost from the epithelium.

**Fig. 2.**
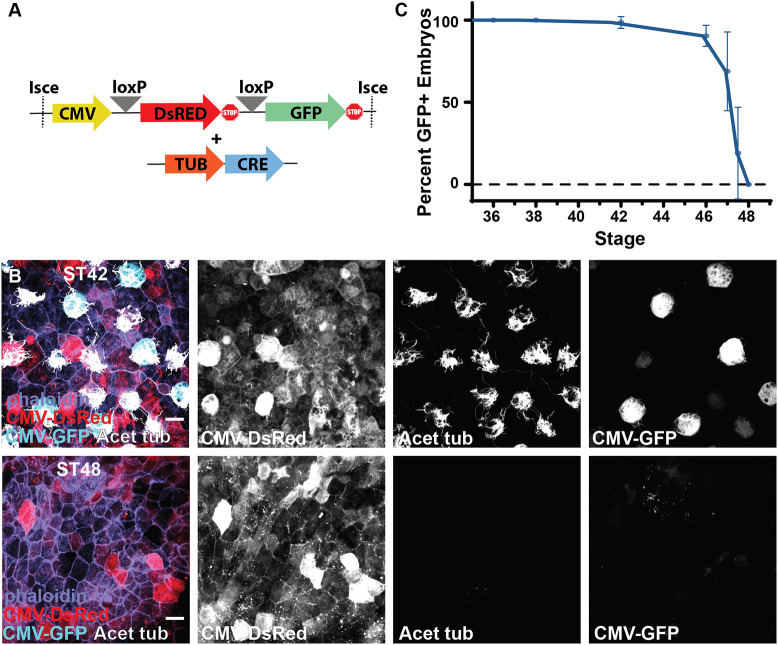
**Lineage tracing of MCC fate.** (A) Experimental design showing lineage-tracing constructs of the CMV DsRed loxP GFP cassette together with the Tub-driven CRE. (B) Lineage-tracing experiment showing the conversion of DsRed to GFP in ST42 MCCs upon expression of CRE specifically in MCCs under control of the Tub promoter, and the loss of MCC-specific CMV expression of GFP despite the maintenance of broad CMV-driven DsRed expression at ST48. (C) Developmental quantification of embryos containing GFP-positive cells in lineage-tracing experiments, *n*=102 transgenic animals over five independent experiments (data are mean±s.d.). Scale bars: 20 µm.

**Fig. 3.**
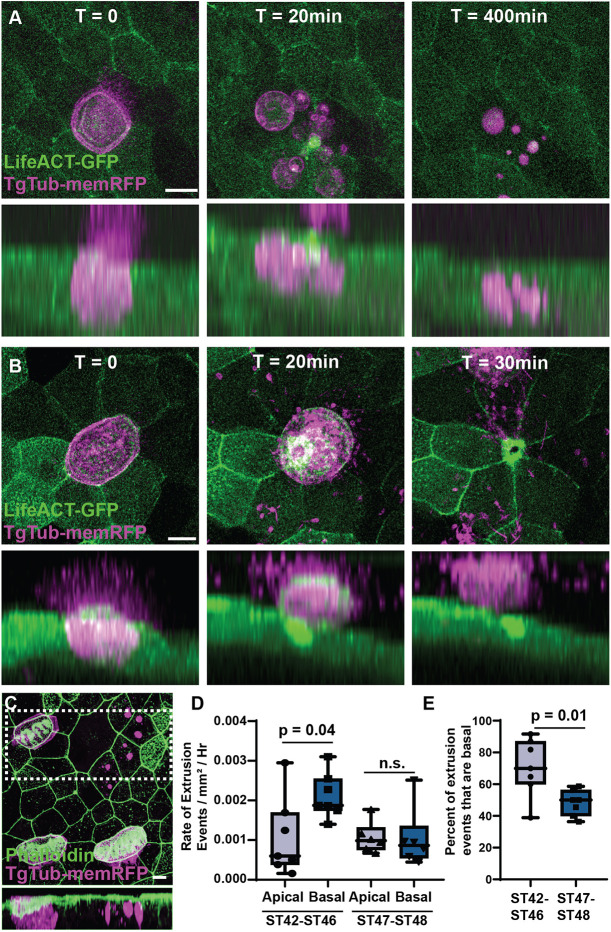
**MCCs are extruded both basally and apically.** (A) Representative frames from a time-lapse movie of TgTub-memRFP embryos injected with LifeACT-GFP showing a MCC undergoing basal extrusion with the RFP-positive cell remnants remaining visible for at least 400 min. Lower panels represents a side projection (see Movie 2). (B) Representative frames from a time-lapse movie showing a MCC undergoing apical extrusion. Lower panel represents a side projection (see Movie 3). (C) Representative example of RFP-positive MCC remnants in fixed tissue stained with phalloidin. Lower panel represents a side projection of boxed area. Scale bars: 10 µm. (D) Quantification of the rate of apical and basal extrusion events using long-term light-sheet imaging between ST42 and ST46 (*P*=0.04), and between ST46 and ST48 (n.s.), *n*=132 extrusion events from five ST42-ST46 embryos, and 69 events from six embryos post ST46-ST48. (E) Percentage of extrusion events that are basal between ST42 and ST46, and ST46 and ST48 (*P*=0.01). Box and whiskers plots represent 25th-75th percentiles (boxes), minimum and maximum values (whiskers), with the line representing the median (see Movie 4).

### Genetic lineage tracing of MCC disappearance

Although the RFP-positive labeling of MCCs and the remnants of dead MCCs using the transgenic line was consistently robust, one could argue that, as MCCs alter their fate, the tubulin promoter shuts off, causing loss of mem-RFP expression from these cells, which would obscure our interpretation that all MCCs are lost. To rule out this possibility, we generated F0 transgenic embryos using a Cre-LoxP-based lineage-tracing cassette in which a CMV promoter drives ubiquitous expression of DsRed with a stop codon, flanked by LoxP sites, and followed by GFP (Fig. 2A) (Werdien et al., 2001; Rankin et al., 2009). In the absence of CRE, CMV drives the expression of DsRed throughout the epithelium without the detectible presence of GFP (Werdien et al., 2001). In contrast, when we introduce CRE specifically in MCCs using the Tub promoter, we see a strong conversion of DsRed to GFP (cyan, Fig. 2B), indicating DsRed excision. Importantly, the MCC-specific GFP expression is still driven by the CMV promoter (Fig. 2B). At ST42, we find many GFP-positive MCCs surrounded by DsRed-positive outer cells, indicating strong expression via the CMV promoter. In contrast, at ST48, we still find strong DsRed expression that indicates CMV promoter activity, but we no longer see GFP-positive cells (Fig. 2B). Although the embryos are transgenic for the lineage tracing construct, the Tub-CRE was injected as plasmid DNA, resulting in mosaic conversion. Consequently, not all MCCs are converted to GFP positive; however, all GFP-positive cells are MCCs, as indicated by the presence of cilia with acetylated tubulin staining (Fig. 2B). Therefore, rather than quantifying the individual number of MCCs, we quantified the number of embryos in which we could identify any GFP-positive cells throughout developmental stages. Although this analysis may not provide an accurate reflection of the total number of MCCs, we regard it as a conservative benchmark for identifying any cells maintained after trans- or de-differentiation. At ST46, most embryos (90%) still have some GFP-positive cells but by ST48 all embryos are devoid of GFP-positive cells (Fig. 2C). These results indicate that although some MCCs may transiently trans- or de-differentiate early, essentially all MCCs are lost from the epithelium by ST48. These results are consistent with our results using TgTub-memRFP (Figs 1B, 2C), which we use throughout the remainder of our experiments.

### Temporal shift from predominantly basal extrusion to an equal mix of apical and basal

During the period of time, when MCC-driven fluid flow decreases between ST38 and ST48, we have used both confocal and long-term light-sheet imaging to observe two distinct forms of cell extrusion. First, as previously mentioned, we observe the remnants of MCCs that have basally delaminated and undergone apoptosis (Fig. 3A,C, Fig. S1 and Movie 1). Cleaved caspase 3 transiently marks apoptotic cells and has previously been shown to mark apoptotic MCCs (Tasca et al., 2021). We find that RFP-positive MCC remnants stain positive for cleaved caspase 3 (Fig. S1), but, importantly, the RFP-positive MCC remnants (vesicular clusters) remain visible for extended periods of time, allowing us to quantify basal cell extrusion in both live and fixed samples (Fig. 3, Fig. S2A-C and Movie 2). Second, we have observed a distinct pool of apically extruding MCCs that are shed from the epithelium as intact cells (Gudipaty and Rosenblatt, 2017; Stewart and Davis, 2019) (Fig. 3B, Movie 3). Using long-term light sheet live imaging, we have quantified the relative percentage of cells that undergo apical versus basal extrusion (Fig. S2D, Movie 4). Between ST42 and ST46 we observe a higher rate of basal extrusion compared with apical extrusion (Fig. 3D), which results in ∼70% of extrusion events being basal (Fig. 3E). However, as development continues, we find a shift, such that, between ST46 and ST48, the rate of apical extrusion and basal extrusion are similar (Fig. 3D) leading to roughly equivalent numbers of apical and basal extrusion events (Fig. 3E). Importantly, this shift towards apical extrusion in later embryos suggests the presence of distinct mechanisms driving apical versus basal extrusion of MCCs.

### Ectodermal caps show the requirement of mesodermal signal for MCC loss

To investigate the mechanisms underlying MCC extrusion, we turned our attention to signaling pathways that govern MCC fate. Previously, Notch signaling has been shown to be a crucial regulator of MCC loss (Tasca et al., 2021). Overriding normal signaling cues by driving the expression of the active Notch intracellular domain (NICD) specifically in MCCs using the Tub promoter leads to an early loss of MCCs via apoptosis. Additionally, blocking Notch signaling with the small molecule inhibitor DAPT leads to an extended maintenance of MCCs. Importantly, the Notch ligands, Jagged, Delta-like1 (Dll1) and Dll4, are expressed in the underlying mesoderm, suggesting that the relevant Notch signal emanates from below (Tasca et al., 2021). To further examine the role of Notch in MCC loss, we generated ectodermal mucociliary organoids from ‘animal caps’ by excising part of the ectoderm before the completion of gastrulation (Sokol and Melton, 1991; Walentek and Quigley, 2017; Weber et al., 1996). Importantly, in the absence of mesoderm, MCCs are maintained in these caps for a considerable time (Fukui et al., 2003). Strikingly, we find that, at 15 dpf (ST50 of embryos), there are still a substantial number of MCCs (Fig. S3A). Even after 1 month, at ST55, we still find numerous MCCs, although some TgTub-memRFP-positive cells contain fewer or even no cilia present, suggesting that MCC maintenance wanes over time (Fig. S3B). As expected, if we express NICD in MCCs using the Tub-promoter in caps, we find that MCCs are again lost, further supporting the importance of Notch signaling and the underlying mesoderm in MCC fate regulation (Fig. S3C-D) (Angerilli et al., 2018; Tasca et al., 2021; Deblandre et al., 1999; Jones and Woodland, 1986; Stubbs et al., 2006). In this context, where embryo growth and elongation is not a contributing factor, these results suggest that Notch signaling generated from the mesoderm is necessary for MCC loss.

### MCCs show age-dependent receptivity to Notch

**Fig. 4.**
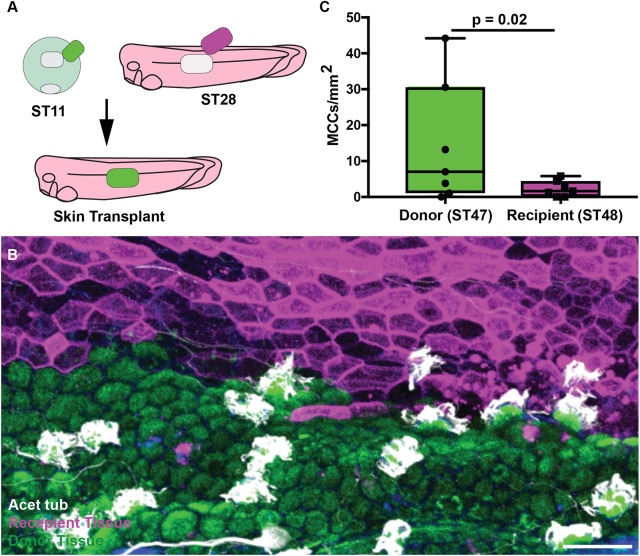
**Skin transplants display age-dependent loss of MCCs.** (A) Experimental design showing the transplantation of ST11 donor skin of *Xla.Tg(CMV:eGFP-OMP25)^Wtnbe^* onto ST28 host skin of a CMV:memb-RFP-injected embryo. (B) Representative image of an area containing both host tissue (red, ST48) and donor tissue (green, ST47) stained for acetylated tubulin (white). Scale bar: 50 µm. (C) Quantification of MCC number in host versus donor tissue, *n*=7 transplants, *P*=0.02. Box and whiskers plot represents 25th-75th percentiles (boxes), minimum and maximum values (whiskers), with the line representing the median.

To determine whether the underlying mesodermal signal is sufficient to drive MCC loss, we performed skin transplant experiments (Mitchell et al., 2009). We replaced small regions of skin from ST28 host embryos injected with mem-RFP with skin from ST11 donor embryos of a transgenic line expressing eGFP-OMP25, a mitochondrial marker under the control of the CMV promoter (Taguchi et al., 2012; Mitchell et al., 2009) (Fig. 4A). We allowed the host embryos to develop to ST48, where most MCCs have been lost. Despite having the same underlying mesoderm, the younger donor tissue (now effectively ∼ST47) maintained many of its MCCs (14.3/mm^2^) at a similar level to ST47 embryos and in contrast to the host tissue (ST48), which was largely devoid of MCCs (2.3/mm^2^, *P*=0.02; Fig. 4B,C). Collectively, these results indicate that the mesoderm provides an instructive cue (via Notch ligands), but that there is a cell-intrinsic, age-based feature that licenses the responsiveness to the Notch signal.

### The MCC transcriptional program is protective against MCC loss

**Fig. 5.**
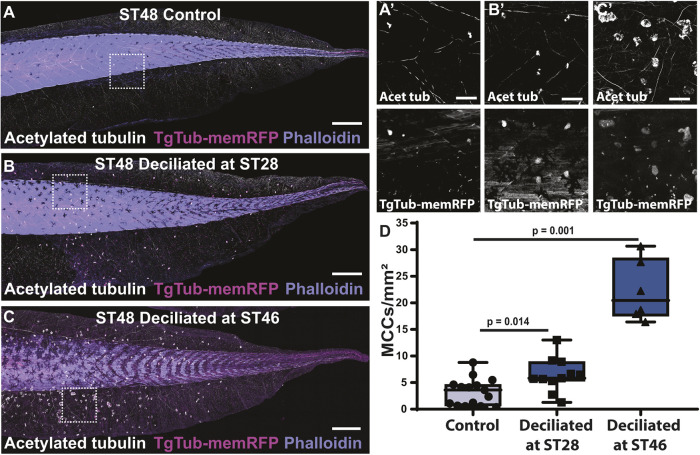
**Deciliation of MCCs reboots the transcriptional program and extends MCC lifespan.** (A-C) Representative whole-tail images of TgTub-memRFP (magenta) embryos stained with acetylated tubulin (white) and phalloidin (purple), showing MCCs in ST48 control embryo (A), as well as ST48 embryos deciliated at ST28 (B) and ST46 (C). (A′-C′) Zoom in of outlined areas in A-C showing acetylated tubulin (top; white) and TgTub-memRFP (bottom; white). Scale bars: 500 μm (A-C); 100 μm (A′-C′). (D) Quantification of MCC number at ST48 in control (*n*≥10 embryos) and embryos that were deciliated at ST28 (*n*≥10 embryos, *P*=0.014) or ST46 (*n*=6 embryos, *P*=0.001). Box and whiskers plot represents 25th-75th percentiles (boxes), minimum and maximum values (whiskers), with the line representing the median (see Fig. S4).

Our results show that the age of MCCs regulates their responsiveness to Notch, which led us to ask whether we could manipulate the fate of MCCs by rebooting their transcriptional program. In the skin of *Xenopus* embryos, it is possible to deciliate MCCs using a brief treatment of high Ca^2+^ and low detergent (Werner and Mitchell, 2013). Importantly, MCCs will completely regrow their cilia within 4 h (Silva et al., 2016). This regrowth is blocked by the protein synthesis inhibitor cycloheximide, suggesting that transcriptional activity and new translation must occur to drive reciliation (Fig. S4). We reasoned that if the ciliogenic program were restarted, this may reset the age-based cell-intrinsic Notch responsiveness. To test this, we deciliated embryos at ST28 and compared MCC number at ST48 with control non-deciliated animals. One might expect that this harsh treatment would make MCCs less healthy and more likely to undergo apoptosis. However, in contrast to controls, the deciliated embryos have a significant, albeit modest, maintenance of MCCs at ST48 (Fig. 5A-C). Importantly, if we carry out this same treatment at ST46 immediately before most of the MCC loss, we see a more robust maintenance of MCCs, indicating a temporal link between boosting the MCC transcriptional program and maintaining MCC lifespan (Fig. 5D). These results suggest that, in order for MCCs to undergo extrusion via the Notch pathway, they must first abandon their MCC transcriptional program. This result is consistent with the transient trans- or de- differentiation model that MCCs are losing their MCC fate before being extruded (Tasca et al., 2021).

**Fig. 6.**
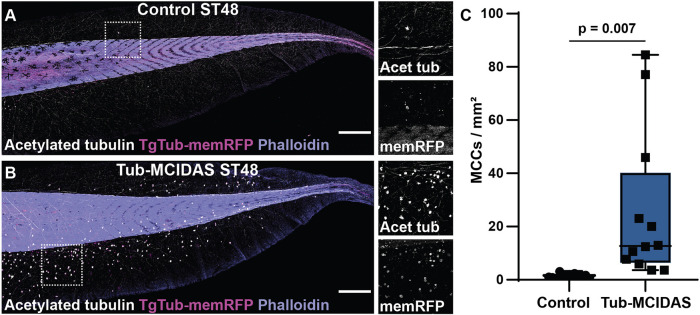
**Maintenance of MCC fate protects against MCC loss.** (A,B) Whole-tail images of control and Tub-MCIDAS transgenic embryos with TgTub-memRFP (red), acetylated tubulin (white) and phalloidin (purple). Scale bars: 500 µm. (C) Quantification of MCC number in control and Tub-MCIDAS embryos at ST48, *n*≥12 embryos, *P*=0.007. Box and whiskers plot represents 25th-75th percentiles (boxes), minimum and maximum values (whiskers), with the line representing the median (see Fig. S5).

The transcriptional program driving MCC differentiation and ciliogenesis is well established (Collins et al., 2021b, Lewis and Stracker, 2021, Walentek, 2022). In *Xenopus* embryos and vertebrate cell culture, the geminin family member MCIDAS has been shown to be essential for driving the formation of MCCs (Kim et al., 2018; Stubbs et al., 2012; Terre et al., 2016). Ectopic expression of MCIDAS in the ectoderm of *Xenopus* embryos converts all epithelial cells into MCCs and the depletion of MCIDAS leads to a loss of MCC specification (Stubbs et al., 2012). MCIDAS expression in *Xenopus* starts before MCC differentiation at ST12 but is largely turned off by ST26, suggesting that it is not essential for the long-term maintenance of the MCC fate (Briggs et al., 2018; Stubbs et al., 2012). However, given the important role of MCIDAS in initiating MCC fate, we reasoned that extending its expression beyond ST26 could have profound effects on MCC fate maintenance and ultimately lifespan. We generated F0 transgenic embryos using a construct that drives overexpression of MCIDAS via the Tub promoter (Tub-MCIDAS) in MCCs. Importantly, tubulin mRNA expression has been shown to be downstream of MCIDAS (Stubbs et al., 2012) and here we show that the TgTub-memRFP transgenic promoter is activated downstream of MCIDAS (Fig. S5), and therefore should generate a positive-feedback loop maintaining MCIDAS expression. Similar to our deciliation experiments, the expression of MCIDAS provides a robust and significant maintenance of the MCC fate at ST48 compared with wild-type epithelia (Fig. 6). These results suggests that the maintenance of the MCC-specification program provides a protective feature against the loss of MCCs. We propose that decommitment to the MCC fate is a licensing event that is required before cells fully respond to the Notch signal and undergo basal extrusion and apoptosis.

### Piezo1 regulation of apical extrusion

**Fig. 7.**
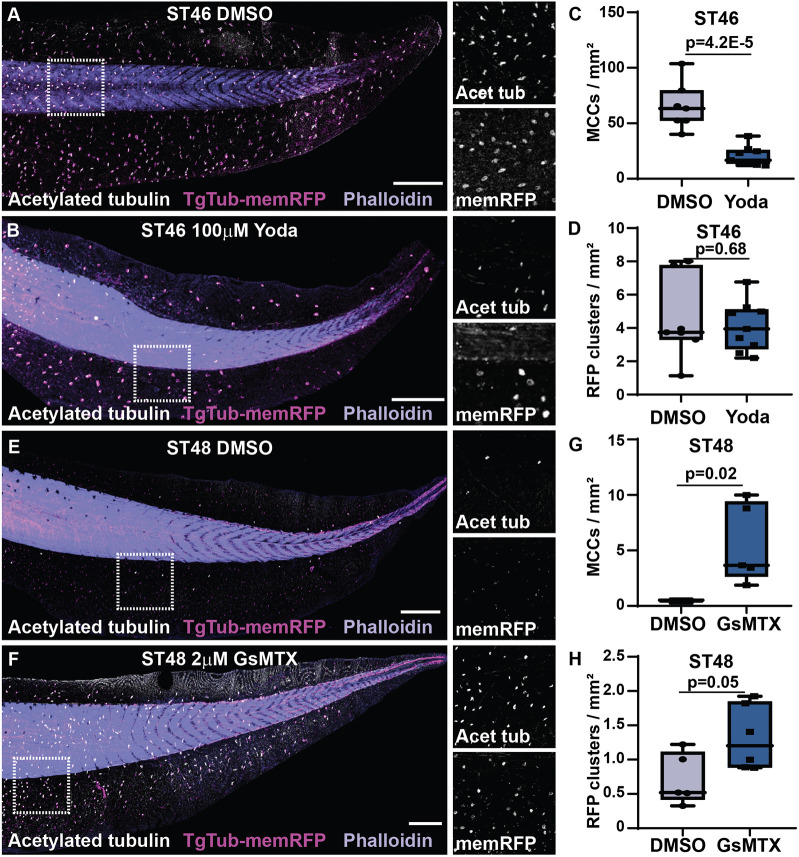
**Piezo1 mechanosensation regulates MCC apical extrusion.** (A-B) Representative images of DMSO-treated (A) and 100 µM Yoda-treated (B) embryos with TgTub-mem-RFP (magenta), acetylated tub (white) and phalloidin (purple). Scale bars: 500 µm. (C) Quantification of MCCs (scored with acetylated Tub) in embryos treated with DMSO and 100 µM Yoda, *n*≥7 embryos, *P*=4.2E-5. (D) Quantification of RFP-positive clusters reflecting apoptotic MCCs in embryos treated with DMSO and 100 µM Yoda, *n*≥7 embryos, *P*=0.68. (E,F) Representative images of DMSO-treated (E) and 2 µM GsMTX-treated (F) embryos with TgTub-memRFP (magenta), acetylated tub (white) and phalloidin (purple). Scale bars: 500 µm. (G) Quantification of MCCs (scored with acetylated Tub) in embryos treated with DMSO and 2 µM GsMTX, *n*≥5 embryos, *P*=0.02. (H) Quantification of RFP-positive vesicles reflecting apoptotic MCCs in embryos treated with DMSO and 2 µM GsMTX, *n*≥5 embryos, *P*=0.05. Box and whiskers plots represent 25th-75th percentiles (boxes), minimum and maximum values (whiskers), with the line representing the median.

The shift in preference from basal towards apical extrusion in older tadpoles could represent a distinct mechanism for eliminating the remaining MCCs that have evaded Notch-driven apoptosis. In some systems of cell extrusion, crowding rather than apoptotic signaling can be an initiator of cell loss (Eisenhoffer et al., 2012; Eisenhoffer and Rosenblatt, 2013; Franco et al., 2019). In this context, the mechanosensory channel Piezo1 has been found to be an important regulator of extrusion. We reasoned that, as development continues, cell crowding and the overall tissue remodeling associated with rapid growth between ST46 and ST48 could be acting as a driver for the second wave of apical MCC extrusion (Zahn et al., 2022; Tasca et al., 2021). To test this, we first treated embryos with the Piezo1 agonist Yoda1. At ST46, when DMSO-treated embryos still maintain a considerable number of MCCs, we found that embryos treated with 100 µM Yoda1 have significantly fewer MCCs (Fig. 7A-C) (Botello-Smith et al., 2019; Lacroix et al., 2018; Syeda et al., 2015). Importantly, we did not find a significant difference in the number of RFP-positive apoptotic vesicles, indicating that the early loss of these MCCs was not at the expense of basally delaminating apoptotic cells (Fig. 7D). This result suggests that activation of Piezo1 is sufficient to drive apical extrusion.

We next tested the Piezo1 inhibitor GsMTX and found that, at ST48, when DMSO-treated embryos were largely devoid of MCCs, there is a significant maintenance of MCCs in embryos treated with 2 µM GsMTX, suggesting Piezo1 is also necessary for apical extrusion (Fig. 7E-G) (Bae et al., 2011). In this context, we do find a slight increase in RFP-positive vesicles that suggests that these populations are at least partially overlapping (Fig. 7H). Although driving early mechanosensory apical loss would not be expected to affect Notch-driven basal loss, the reverse is not as likely. Given that these modes of extrusion overlap, we would expect that blocking mechanosensory loss via GsMTX would lead to more cells that are available to be responsive to Notch later and therefore driven towards basal extrusion. Overall, these results suggest that there are two independent mechanisms for MCC extrusion: an apoptotic basal extrusion driven by Notch signaling and a live apical extrusion driven by a Piezo1-mediated mechanosensory mechanism.

## DISCUSSION

With each MCC containing ∼150 cilia that each beat at ∼15 Hz, the epithelial fluid flow that MCCs generate is extremely energetically taxing to the embryo. When flow is no longer needed due to the swimming motion of the tadpole, there would presumably be a strong selective pressure to remove these cells, as is suggested by the conserved loss of MCCs in other amphibians (Smith et al., 1988; Kessel et al., 1974; Smith et al., 1990). Additionally, tadpoles use the lateral line system which detects vibrations in the water for orientation and predation escape, which would presumably be adversely affected by cilia generated fluid flow. Notch signaling is a crucial regulator of ciliated epithelia both during development as well as during tissue remodeling after insult (Deblandre et al., 1999; Lafkas et al., 2015; Liu et al., 2007; Rock et al., 2011; Tasca et al., 2021). During *Xenopus* MCC loss, Notch is a crucial signal that pushes MCCs towards apoptosis and basal extrusion. Here, we propose that the receptivity to the Notch signal is regulated by the overall MCC transcriptional state of the cell. This is based on three experiments. First, skin transplants of younger skin do not respond with the same kinetics as older host tissue. Second, deciliated MCCs reboot their MCC transcriptional program to rebuild their cilia, and this offers resistance to the Notch signal, extending MCC lifespan. Finally, continued expression of the MCC transcriptional regulator MCIDAS maintains the MCC transcriptional program and extends MCC lifespan. Our data suggest a balance between MCC fate maintenance and the ability to respond to the Notch signal via apoptosis and basal extrusion. We propose a licensing mechanism where MCCs must first decommit from their fate before becoming responsive to Notch-driven apoptosis. In all of our experiments, the Notch ligand should be consistently present, suggesting that the change as MCCs decommit is likely due either to a change in Notch receptor expression levels or to Notch receptor subtype specificity and sensitivity. This is consistent with our animal cap data, as well as previously published work that the expression of the constitutively active NICD in MCCs is sufficient to drive apoptosis (Tasca et al., 2021). However, the previously proposed idea that MCCs stably transdifferentiate into long-lived mucus secreting cells is not well supported by our current data. Our lineage-tracing experiments indicate that all MCCs are absent by ST48, suggesting that trans- or de-differentiation is transient. Modulation of cell fate, however, does appear to be an important component of MCC loss, as we propose that the decommitment to the MCC lineage is integral to their extrusion. Consistent with this, miR-34/449 is part of the MCC transcriptional program and is known to negatively regulate the Notch pathway, which could explain why decommiting from the MCC fate leads to a change in Notch sensitivity (Song et al., 2014; Marcet et al., 2011; Walentek et al., 2014).

Cell extrusion is an important mode of tissue remodeling that is crucial for removing unwanted, less competitive or dying cells (Eisenhoffer and Rosenblatt, 2013; Gudipaty et al., 2018; Mitchell and Rosenblatt, 2021). Cell extrusion has been widely studied and there are an extensive number of tissues that have been shown to exhibit this behavior. What makes the extrusion of MCCs distinct is that a specific cell type that is interspersed across the epithelium is lost within a defined developmental period by two distinct yet overlapping mechanisms. Even more unusual is that the direction of extrusion differs between these two mechanisms. In most systems, cell extrusion is unidirectional and occurs only bidirectionally in disease or experimental conditions (Eisenhoffer et al., 2012; Monier et al., 2015; Nanavati et al., 2020; Villars and Levayer, 2022). Yet here we show that MCCs naturally undergo both Notch-driven basal extrusion and Piezo1-regulated apical extrusion during an overlapping developmental window. We propose that MCCs will respond to the Notch signal and apoptose if they have decommitted from their MCC fate. If they do not respond, owing to differences in age (e.g. some MCCs are born later than others), signaling environment (e.g. Jak/Stat and thyroid hormone) or a potentially unfavorable position relative to the source of the Notch ligand, they will be driven to apical extrusion via mechanosensation. One possibility is that the apical extruding cells are undergoing live-cell extrusion, which is supported by the observation that these cells maintain functional cilia until the point of extrusion (e.g. Fig. 3B, Movie 3) (Eisenhoffer et al., 2012; Eisenhoffer and Rosenblatt, 2013). However, it remains possible that mechanosensation induces a distinct apoptotic event that is missed by our current analysis.

Mechanosensory-driven cell extrusion has been described in a variety of tissues, yet the extruding cells are typically thought to be in an energetically unfavorable position (e.g. the edge of the tissue). Our Piezo1 data add to a growing list of diverse functions for Piezo1, which also includes regulating the scaling of MCC size to centriole number; however, how Piezo1 regulates MCC size early in development while regulating extrusion later remains unknown (Kulkarni et al., 2021). MCCs are distinct in that they maintain a significant cytoskeletal architecture at their apical surface that could provide a distinct environment for mechanosensation and facilitate apical extrusion. Another possibility is that as MCCs decommit from their fate, they become less energetically distinct from their neighbors and represent an unhealthy and less-competitive cell that is susceptible to extrusion. Alternatively, the remaining MCCs could maintain their fate and their cytoskeletal architecture, and, as such, be targeted for extrusion based on their energetically distinct cellular profile. Distinguishing between these alternatives as well as determining whether Piezo1 activity is required within MCCs or the surrounding cells will be an important aspect of future work.

## MATERIALS AND METHODS

### Transgenic *Xenopus* and embryo injections

All *Xenopus* experiments were performed using previously described techniques (Werner and Mitchell, 2013). *In vitro* fertilizations were performed using standard protocols (Sive et al., 1998, 2007b, 2010) that have been approved by the Northwestern University Institutional Animal Care and Use Committee (IS00006468). Transgenic *Xenopus* that express membrane-bound RFP driven by the tubulin promoter [Xla.Tg(tuba1a:MyrPalm-mRFP)^NXR^] were obtained from the National *Xenopus* Resource Center (NXR; XL-LINE-1468). For skin transplants, transgenic *Xenopus* that express eGFP-tagged OMP driven by the CMV promoter [Xla.Tg(CMV:eGFP-OMP25)^Wtnbe^], that allows constitutive GFP expression in mitochondrial membranes were obtained from NXR (Taguchi et al., 2012). Wild-type or transgenic embryos were injected at the two- or four-cell stage with 40-250 pg mRNA or 10-20 pg of plasmid DNA.

### Plasmids and mRNA

In this study, we employed a CMV promoter to drive constitutive expression of GFP or RFP as tracers in all cell types, whereas MCC-specific expression was driven by an α-tubulin (aTub) promoter, as previously described (Stubbs et al., 2006).

#### I-sceI-CMV-LoxP-DsRed-LoxP-GFP

To generate the I-sce-CMV-DsRed-LoxP-GFP cassette, CMV-LoxP-DsRed-LoxP-GFP was amplified from pLV-CMV-LoxP-DsRed-LoxP-GFP (Addgene 65726) (Zomer et al., 2015) and inserted into an I-sceI vector, a gift from Marko Horb at the Marine Biological Laboratory (MBL; Woods Hole, MA, USA), by Gibson Assembly.

#### pCS2-aTub-Cre2 and I-sceI-Tub-Cre2

The CMV promoter of pCS.Cre2 (Addgene 31308) (Ryffel et al., 2003) was replaced with an α-tubulin promoter by SalI(5′)/HindIII(3′). To insert into the I-sceI vector, aTub-Cre2 was cut out and ligated into an I-sceI vector using SalI(5′)/KpnI(3′).

#### pCS2-aTub-NICD-GFP

Notch intracellular domain (NICD) (Deblandre et al., 1999) was inserted into the pCS2-aTub-GFP construct using ClaI(5′)/XbaI(3′).

#### I-sce-aTub-GFP-MCIDAS

The CMV promoter of pCS2-MCIDAS (Stubbs et al., 2012) was replaced with aTub-GFP by SalI(5′)/EcoRI(3′) to generate pCS2-tub-GFP-MCIDAS. aTub-GFP-MCIDAS was then inserted into the I-sceI vector using the SalI(5′)/KpnI(3′) sites.

pCS2-memb-RFP (Stubbs et al., 2006) was used for skin transplants, and mRNA of LifeAct-GFP (Werner and Mitchell, 2013) was synthesized with the Sp6 mMessage Machine kit (Life Technologies, AM1340) and purified by RNeasy MiniElute Cleanup Kit (Qiagen, 74204).

### I-sceI transgenesis

To generate transgenic embryos, we used the I-sceI meganuclease-mediated method, as previously described (Ishibashi et al., 2012; Ogino and Ochi, 2009; Pan et al., 2006). I-sceI-CMV-LoxP-DsRed-LoxP-GFP construct was incubated with I-sceI at 37°C for 40 min and embryos were injected with 40 pg of DNA and 0.004 U of I-sceI per embryo at the one-cell stage for 1 h immediately followed by de-jelling. To optimize transgenesis efficiency and minimize toxicities from a high amount of DNA and enzyme injections, we used only one I-sceI construct for the CMV-LoxP-DsRed-LoxP-GFP cassette (Thermes et al., 2002). Embryos were then allowed to heal and pCS2-aTub-Cre was injected at the four-cell stage. After transgenesis at ST38, the Leica M165 FC dissecting microscope was used to isolate embryos for lineage tracing that were positive for GFP expression, indicating a DsRed-to-GFP conversion in MCCs. The percentage of these embryos that remained GFP positive was scored at various developmental stages.

### Fluid flow measurement

Fluorescent microspheres were used to visualize cilia-driven fluid flow at different stages of development, as previously described using the Leica M165 FC dissecting microscope connected to the Casio Exilim 60fps mounted digital camera (Werner and Mitchell, 2013). In brief, fluorescent microspheres (F8836, Invitrogen) were resuspended in 0.1×MMR containing 0.5% glycerol. Embryos were placed in 0.02% tricaine+0.1×MMR and fluorescent beads were added dropwise to the embryo. The relative velocity of the fluid flow was calculated by determining the displacement of individual microspheres over the surface of the embryo by using simple particle tracking software available on FIJI (Schindelin et al., 2012).

### Immunostaining

Embryos were fixed with 3% PFA/PBS and blocked in 10% heat-inactivated goat serum (HIGS)/PBS after washing with PBST. To visualize cilia, embryos were incubated with mouse anti-acetylated α-tubulin (T7451; Sigma-Aldrich, 1:500) or anti-cleaved caspase 3 (9664, Cell Signaling, 1:100) in 5% HIGS/PBST for 1 h at room temperature. Cy-2- or Cy-5-conjugated goat anti-mouse secondary antibodies (Thermo Fisher Scientific) were then used at a 1:750 dilution in 5% HIGS/PBST. To visualize actin and nuclei, Phalloidin 650 (PI21838, Invitrogen, 1:300) and DAPI (62248, Thermo Fisher Scientific, 1:500) were used. After staining, embryo tails were mounted between two coverslips as previously described (Werner and Mitchell, 2013) using Fluoro-Gel (1798510, Electron Microscopy Sciences).

### Microscopy and quantification

Fixed samples were imaged using the Nikon A1R laser scanning confocal microscope using a 60× oil Plan-Apochromat objective lens with a 1.4 NA. For whole tail imaging, a 20× water Plan Fluor objective lens with a 0.75 NA with Large Image function was used. Maximum intensity projections of *z*-stacks were analyzed using either Nikon NIS Elements or FIJI (Schindelin et al., 2012). Live imaging of apical and basal extrusion was performed using the Nikon A1R laser scanning confocal microscope with a 60× oil Plan-Apochromat objective lens with a 1.4 NA. Long-term imaging for quantification of apical and basal extrusion was performed on TgTub-memRFP embryos using a Nikon Ti2 microscope equipped with a Mizar TILT light sheet and a photometric prime 95B camera, using a 10× objective (N.A. 0.3) or 100× silicone objective (N.A. 1.35). For live imaging, embryos were anesthetized and restricted from moving forward but were free ‘floating’ and were not otherwise mounted or embedded so as not to restrict apical extrusion. Images were analyzed using FIJI software (Schindelin et al., 2012) to quantify the number of extrusion events (both apical and basal) that occur per mm^2^ per h. Quantifications throughout this work were carried out using either NIS-Elements or FIJI software and error bars represent s.d. Statistics for all direct comparisons were performed using Students *t*-test (two-tailed) or a non-parametric test if the normality of the data was violated. All quantifications were performed on data collected from at least four embryos (as listed in the figure legends) collected from at least two separate experimental fertilizations. Box-and-whisker plots show the median, upper and lower quartiles (box) and the minimum and maximum values.

### Ectodermal caps

Embryonic ectodermal caps were isolated at ST10 in 0.5X MMR+gentamicin, following a previously described protocol (Sive et al., 2007a). Caps were then transferred to fresh 0.5× MMR+gentamicin to recover. After healing, caps were treated with 0.5 ng/ml of activin A (A4941-10, Sigma-Aldrich) in 0.5× MMR+gentamicin for 1 h to promote development to epidermis (Ariizumi et al., 1991). Caps were transferred to Leibovitz L-15 cell culture medium+Steinberg+1% BSA+antibiotics for long-term culture (Ariizumi et al., 2017; Fukui et al., 2003). Uncapped embryos were used as a stage reference. Once caps are grown for 2 weeks (∼ST50) or 1 month (∼ST55), they were fixed in 3% PFA/PBS and stained using antibodies.

### Skin transplants

Embryonic skin transplants were performed as previously described (Mitchell et al., 2009; Werner and Mitchell, 2013). Briefly, a small region of ectoderm from a donor embryo [Xla.Tg(CMV:eGFP-OMP25)^Wtnbe^] at ST11 was removed with a fine hair then transplanted onto the recipient embryo at ST28 injected with pCS2-memb-RFP after removing a similar patch of outer skin layer. All transplant experiments were performed in Danilchik's buffer+0.1% BSA+0.02% tricaine. Transplanted tissue was then held in place by gently pressing down with a small piece of glass coverslip using silicone grease to secure its position for ∼1-2 h. After healing, the coverslip was gently removed, and embryos were grown in 0.1× MMR until the desired stage was reached.

### Deciliation

Using a previously described method (Werner and Mitchell, 2013), embryos were deciliated at various developmental stages in 0.1× MMR+gentamicin containing 75 mM calcium and 0.02% NP40. Embryos were incubated in this solution for ∼20-40 s and then washed several times in 0.1× MMR+gentamicin. Several control embryos were immediately fixed in 3% PFA/PBS following treatment to confirm successful deciliation. The remaining embryos were incubated in 0.1× MMR+gentamicin until reaching the desired stage, then fixed in 3% PFA/PBS for analysis.

### Drug treatments

Yoda1 (Fisher, 558610) was used to activate Piezo1 mechanosensory channels, whereas GsMTX (MedChemExpress, HY-P1410) was used to inhibit Piezo1 channels. Embryos were incubated in the presence of DMSO (vehicle), 100 µM Yoda (in DMSO) or 2 µM GxMTX (in DMSO) beginning at ST28 of development until they reached the desired stage. Embryos were then fixed in 3% PFA/PBS.

## Supplementary Material

10.1242/develop.201612_sup1 Supplementary information
