# Supplementary material for: Bidirectional multiciliated cell extrusion is controlled by Notch-driven basal extrusion and Piezo1-driven apical extrusion
Source: Development. 2023 Sep 1;150(17):dev201612. doi: 10.1242/dev.201612 (PMC10482390; doi:10.1242/dev.201612)
Supplement: Supplementary information [file develop-150-201612-s1.pdf]

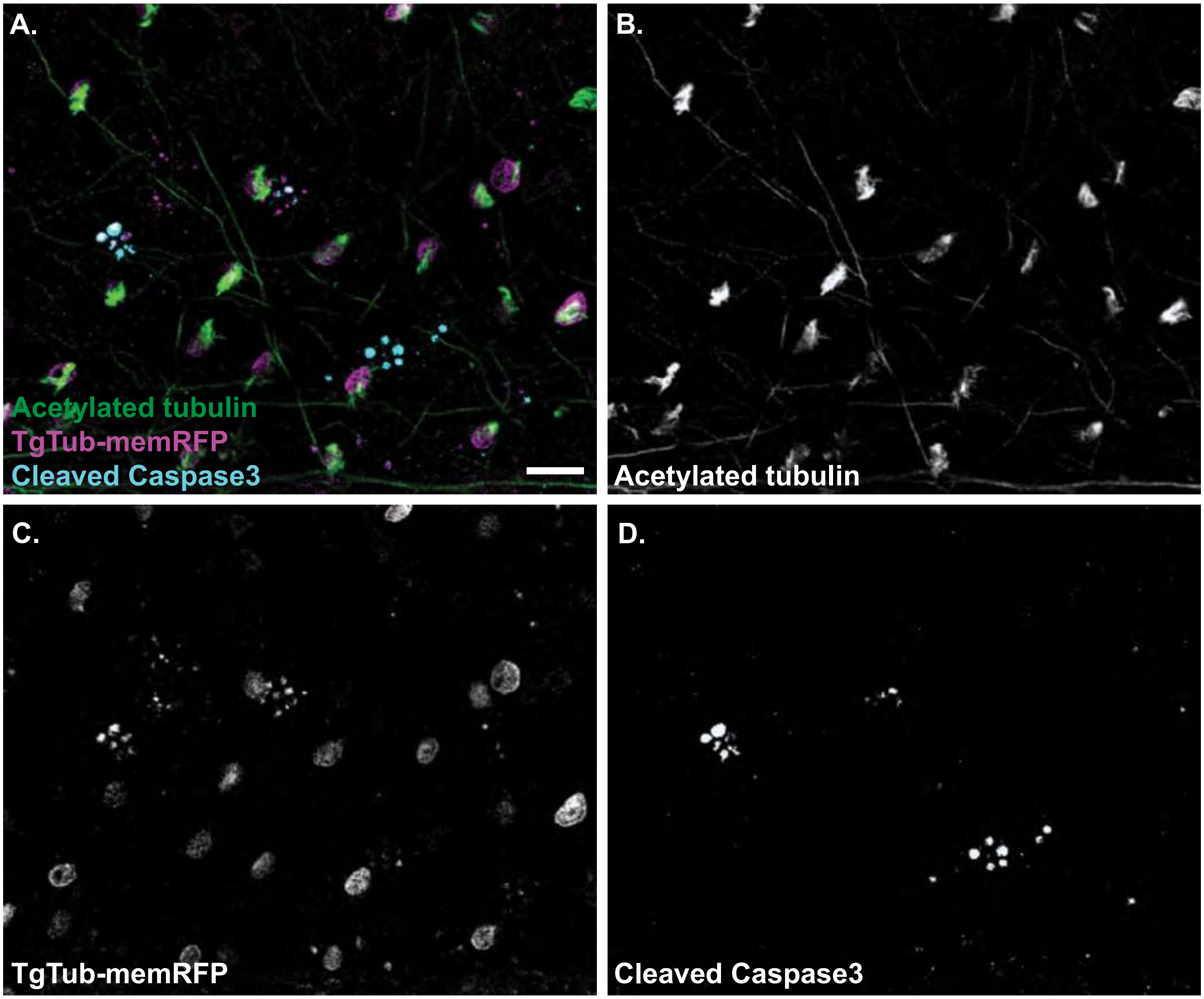

Fig. S1. Basal extrusion of apoptotic cells. (A-D) ST47 TgTub-memRFP embryo showing MCCs and RFP + MCC remnants (magenta (A), white (C)) stained with anti- acetylated tubulin (green (A), white (B)) to mark cilia and anti- Cleaved Caspase 3 (cyan (A), white (D)) to mark apoptotic cells. Scale is 50 μm.

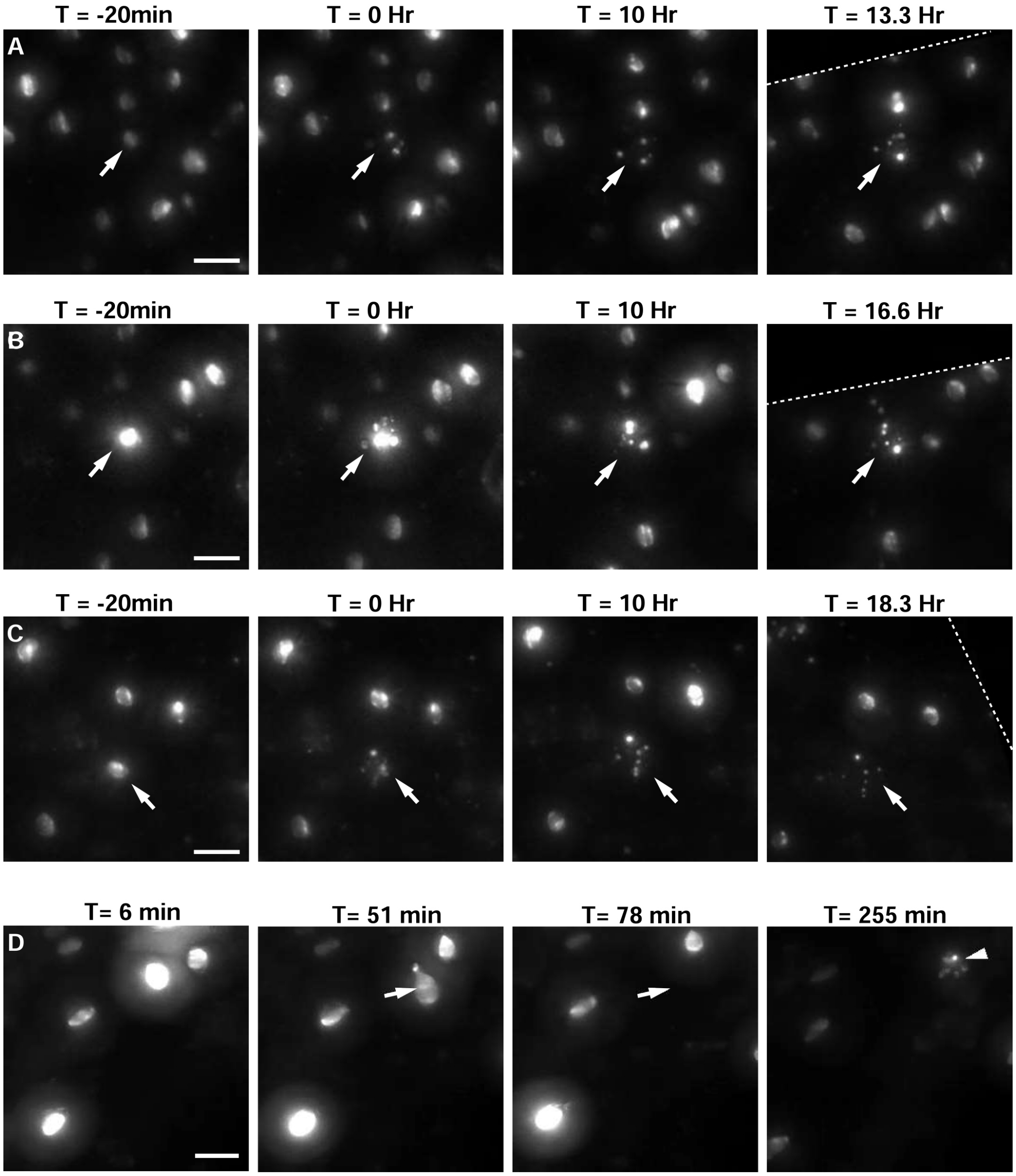

Fig. S2. MCC apical and basal extrusion and RFP vesicle maintenance. (A-C) Representative examples from long-term light-sheet movies showing maintenance of RFP posit ve vesicles (arrows) after basal extrusion for 13.5 Hrs (A), 16.6 Hrs (B) and 18.3 Hrs (C.also see Movie 52) when the cells/embryo eventually moved out of frame (out of frame depicted via dotted line). (D) Light-sheet imaging showing MCC disappearance during apical extrusion (arrow) and RFP cluster formation during basal extrusion (arrowhead;see Movie 54). Scale is 50μm.

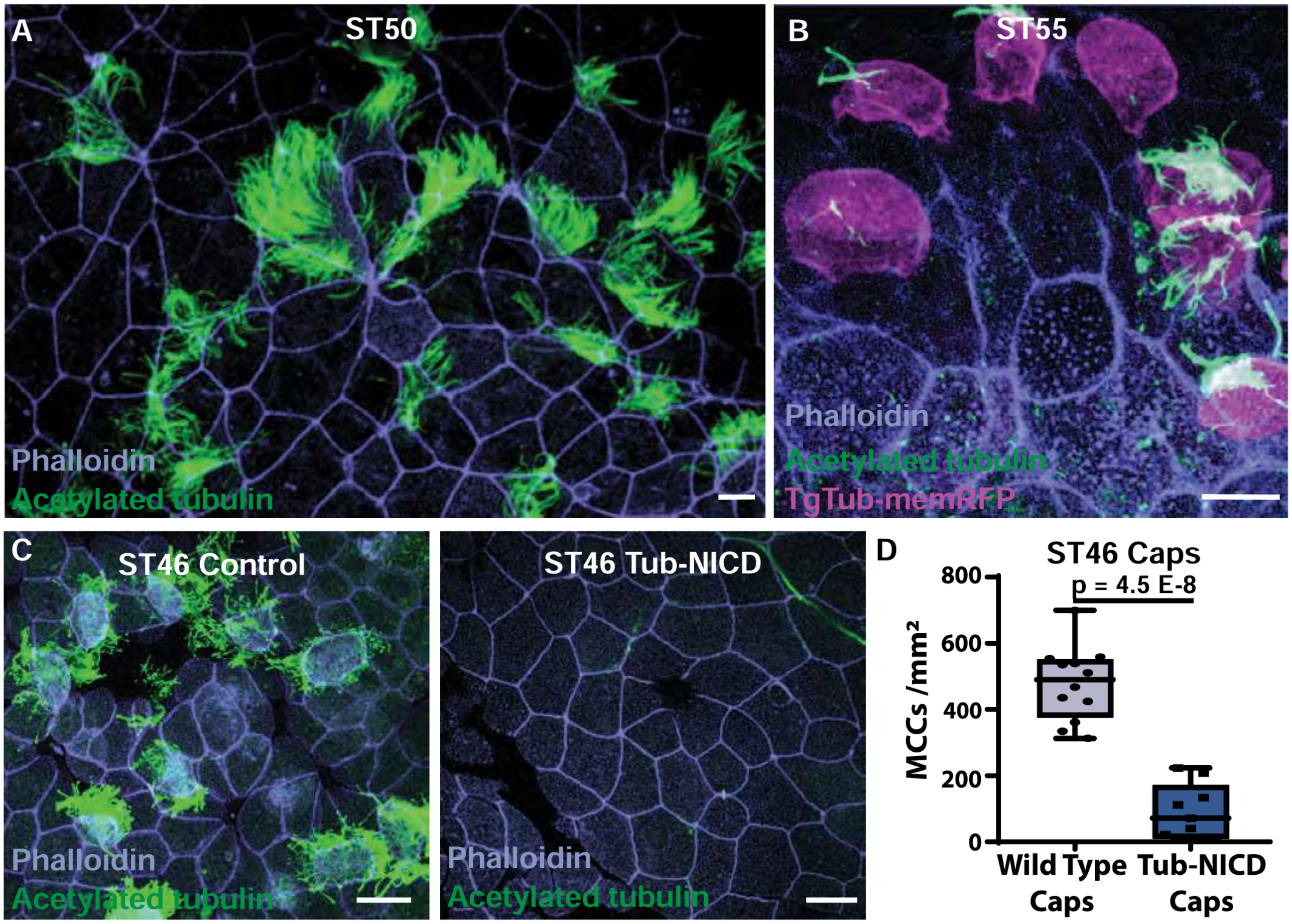

Fig. S3. Mesodermal Notchis required for MCCloss. (A) Day 15 ST50 animal cap that still maintains a large number of MCCs. (B) Day 30 ST55 animal cap from a TgTub-memRFP embryo that shows the presence of multiple MCCs but also the loss of cilia and MCC maintenance in some RFP positive cells. (C) Comparison of MCCs in caps from WT embryos and embryos injected with Tub-NICO.(D) Quantification of MCCs in caps with and without Tub-NICO (n > 3 caps; p = 4.SE-8). Scale bar is 20μm.

Development • Supplementary information

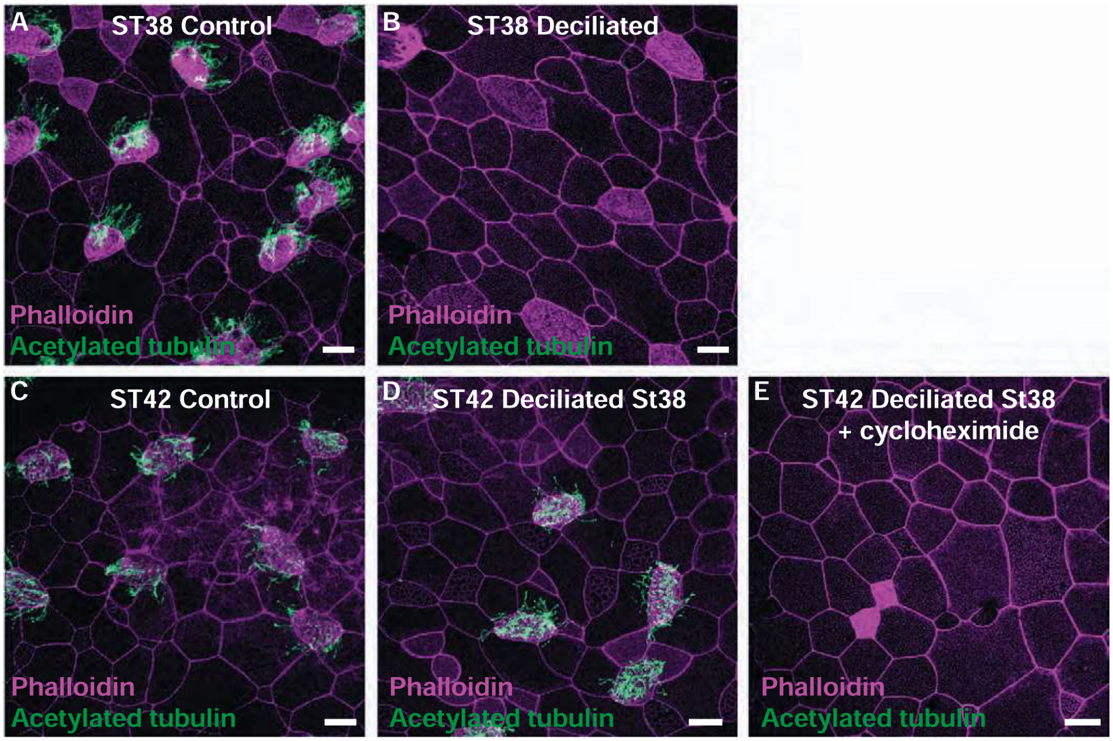

Fig. S4. Multiciliated cell deciliation. (A-E) Representative images of a embryos stained with phalloidin (magenta) and acetylated tubulin (green). (A-B) Representative images of a con- trol embryo at ST38 (A) and a ST38 embryo directly after deciliation (B). (C-E) Representative images of a controlembryo at ST42 (C), a ST42 embryo that was deciliated at ST38 (D), and an embryo that was deciliated at ST38 but that was also treated with cycloheximide (E). Scale is 20μm.

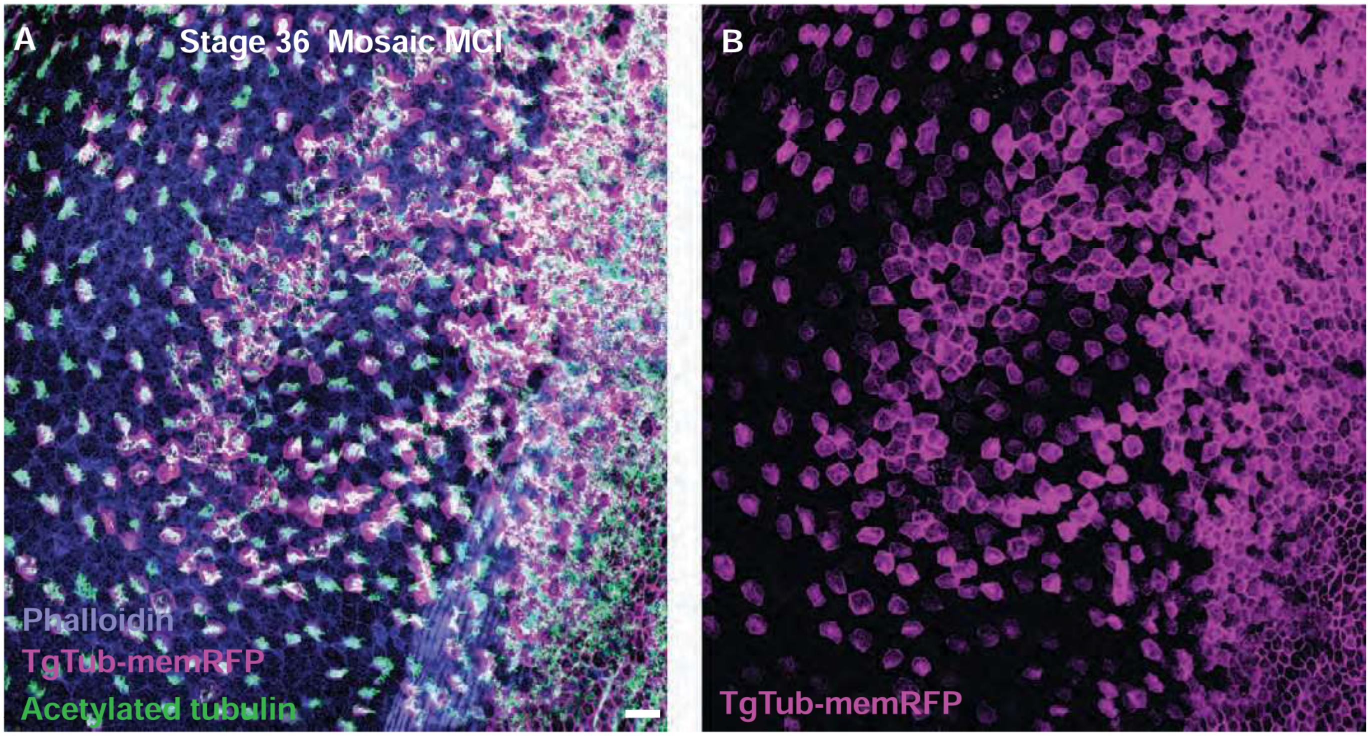

Fig. S5. Tub promoter activity is downstream of MCIDAS. (A-B) ST36 embryo mosaically injected with the MCC-inducing factor MCIDAS, showing broad expression of the TgTub-mem-RFP (magenta) in ectopic MCCs labeled with acetylated tubulin (green) with phalloidin staining (purple) (A) and the same image showing only the ectopic TgTub driven mem-RFP (B). Scale is 50μm.

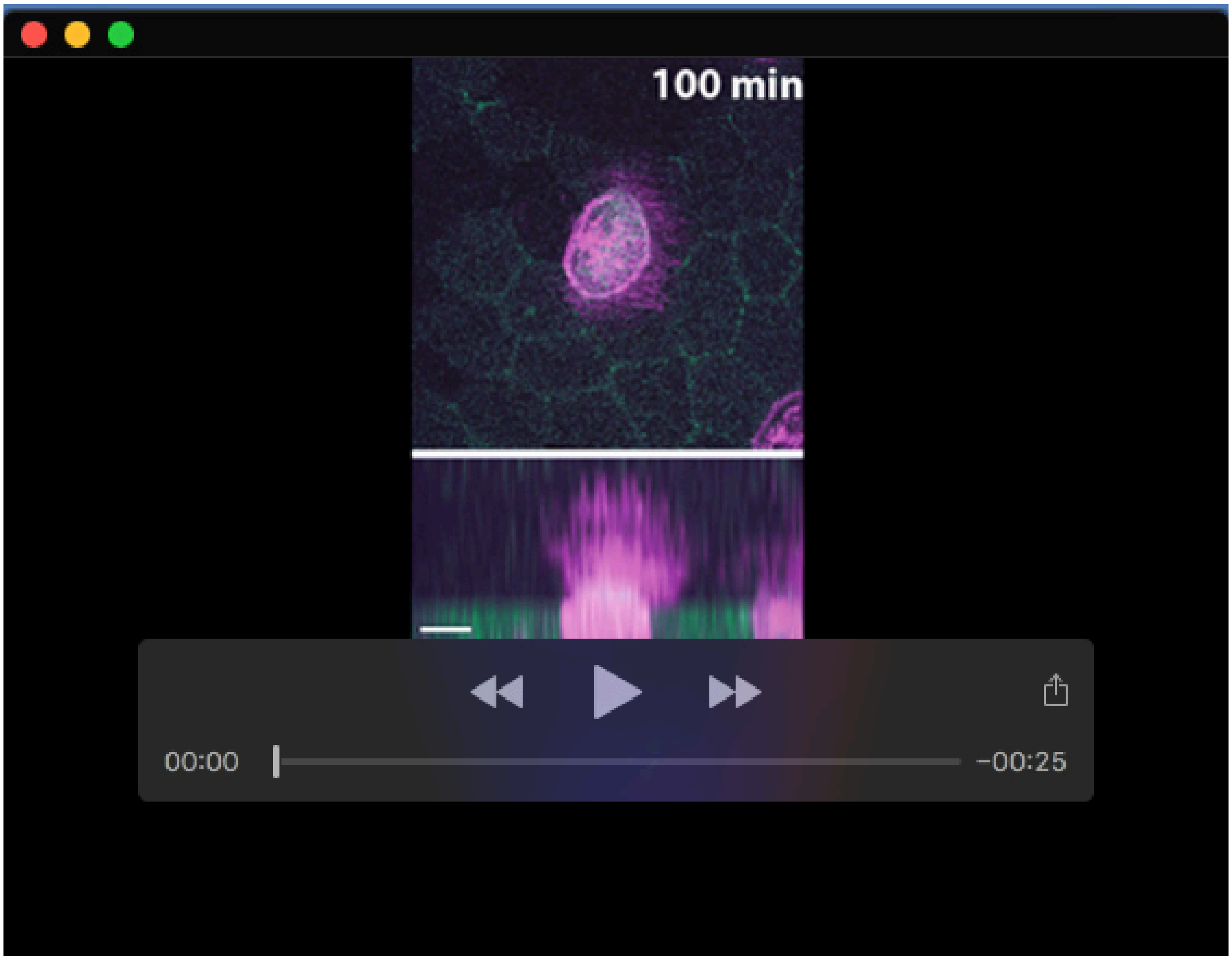

Movie 1. MCC basal extrusion. Z and side projections of the data in Figure 3A of an embryo expressing TgTub-memRFP and LifeACT-GFP showing a cell basally extruding. Movie is 35 frames with images taken every 20 minutes. Scale is 10μm.

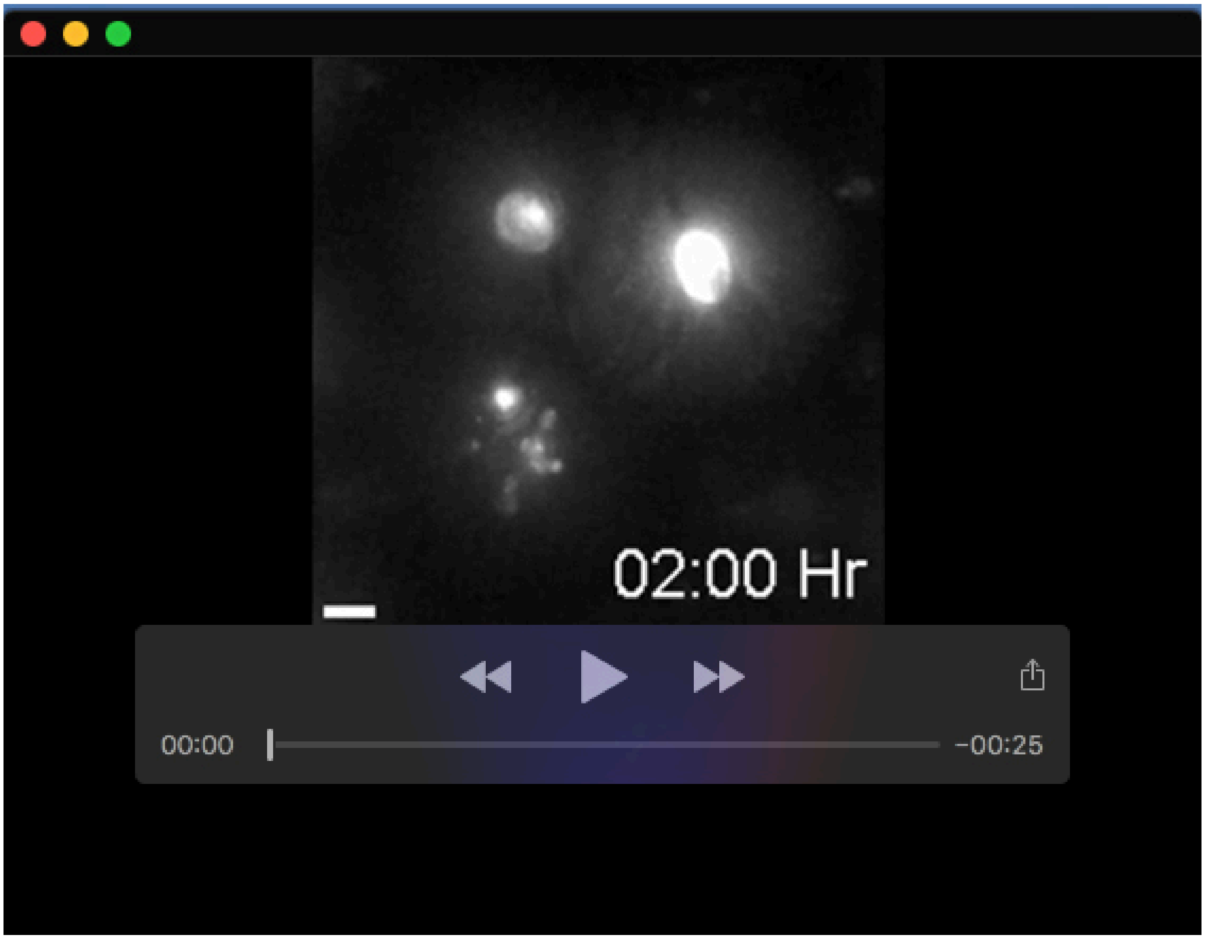

Movie 2. Maintenance of RFP positive vesicles. Cropped image from a low resolution (10X) long-term light-sheet movie that shows an MCC undergoing basal extrusion and apoptosis , with the RFP positive cluster remaining visible for ~18HRs until the movie moves out of frame. Movie is 54 frames taken every 20 minutes. Scale is 20µm.

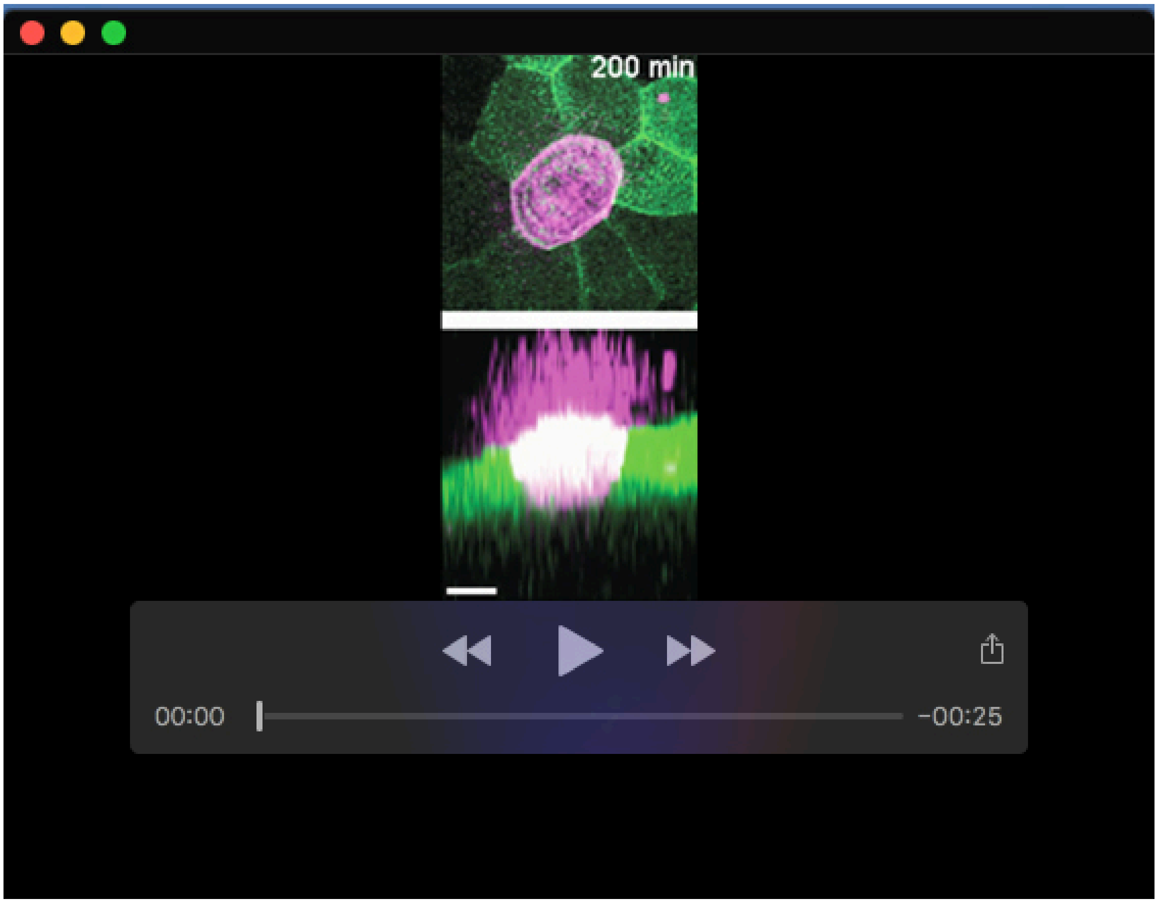

Movie 3. MCC apical extrusion. Z and side projections of the data in Figure 3B of an embryo expressing TgTub-memRFP and LifeACT-GFP showing a cell apically extruding. Movie is 25 frames with images taken every 10 minutes. Scale is 10µm.

Development • Supplementary information

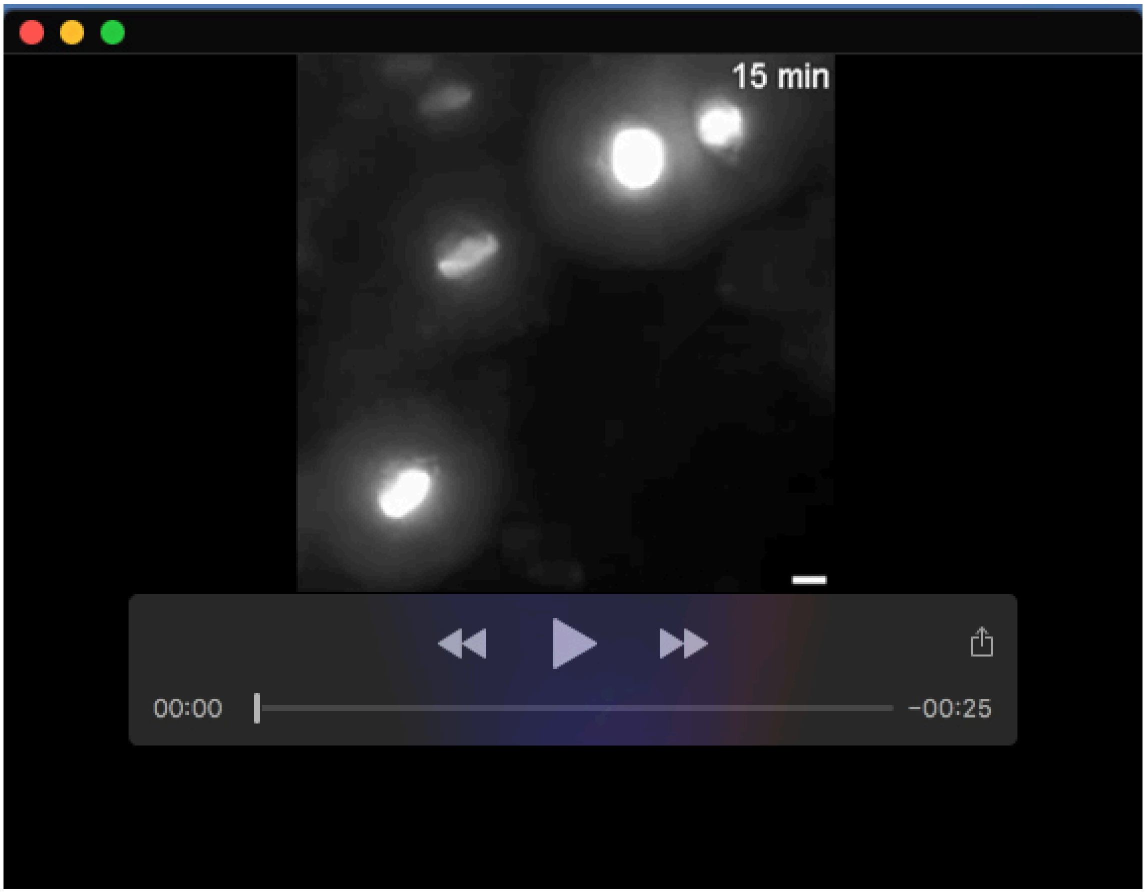

Movie 4. MCC apical and basal extrusion. Cropped image from a low resolution (10X) long- term light-sheet movie that shows how we quantified apical extrusion (arrow frame 15) and basal extrusion (arrowhead frame 81). Movie is 100 frames with images taken every 3 minutes. Scale is 20µm.

Development • Supplementary information
