# Supplementary material for: Bidirectional multiciliated cell extrusion is controlled by Notch-driven basal extrusion and Piezo1-driven apical extrusion
Source: Development. 2023 Sep 1;150(17):dev201612. doi: 10.1242/dev.201612 (PMC10482390; doi:10.1242/dev.201612)
Supplement: Supplementary file 1 [file develop-150-201612_review_history.pdf]

Bidirectional multiciliated cell extrusion is controlled by Notch-driven basal extrusion and Piezo1-driven apical extrusion

Rosa Ventrella, Sun K. Kim, Jennifer Sheridan, Aline Grata, Enzo Bresteau, Osama A. Hassan, Eve E. Suva, Peter Walentek and Brian J. Mitchell

DOI: 10.1242/dev.201612

Editor: Thomas Lecuit

Review timeline

Original submission:	12 January 2023
Editorial decision:	28 February 2023
First revision received:	27 June 2023
Accepted:	11 August 2023

Original submission

First decision letter

MS ID#: DEVELOP/2023/201612

MS TITLE: Bidirectional multiciliated cell extrusion is controlled by Notch driven basal extrusion and Piezo 1 driven apical extrusion.

AUTHORS: Rosa Ventrella, Sun K. Kim, Jennifer Sheridan, Aline C. Grata, Enzo Bresteau, Osama A. Hassan, Evelyn E. Suva, Peter Walentek, and Brian Mitchell

I have now received all the referees' reports on the above manuscript, and have reached a decision. The referees' comments are appended below, or you can access them online: please go to BenchPress and click on the 'Manuscripts with Decisions' queue in the Author Area.

As you will see, the referees express considerable interest in your work, but they also point out several issues that require further clarification and in a few instances further experimental support. It is not necessary to do new experiments to address new points. However, please focus on those points that require further investigation to ascertain your interpretations. If you are able to revise the manuscript along the lines suggested, I will be happy receive a revised version of the manuscript. Your revised paper will be re-reviewed by one or more of the original referees, and acceptance of your manuscript will depend on your addressing satisfactorily the reviewers' major concerns. Please also note that Development will normally permit only one round of major revision. If it would be helpful, you are welcome to contact us to discuss your revision in greater detail. Please send us a point-by-point response indicating your plans for addressing the referee’s comments, and we will look over this and provide further guidance.

Please attend to all of the reviewers' comments and ensure that you clearly highlight all changes made in the revised manuscript. Please avoid using 'Tracked changes' in Word files as these are lost in PDF conversion. I should be grateful if you would also provide a point-by-point response detailing how you have dealt with the points raised by the reviewers in the 'Response to Reviewers' box. If you do not agree with any of their criticisms or suggestions please explain clearly why this is so.

© 2023. Published by The Company of Biologists under the terms of the Creative Commons Attribution License (https://creativecommons.org/licenses/by/4.0/).

<u>Reviewer 1</u>

Advance summary and potential significance to field

The paper by Ventrella et al. describes the process of multiciliated cells (MCCs) extrusion from the mucociliary epithelium of Xenopus embryos. The authors provide exciting evidence of bidirectional extrusion– basal and apical, driven by Notch and Piezo, respectively. While its main findings will certainly interest many in the broad audience of readers of this journal, I feel that a significant amount of work needs to be put into this publication for it to meet the standards of a journal like Development.

Comments for the author

1. The authors should describe in further detail the cellular mechanisms that govern bidirectional cell extrusion. This is the focus of the paper, yet largely not addressed by the authors. Many important questions arise when thinking about the cell extrusion process. Is it cell autonomous, or there is the contribution of the neighboring cells? How are actin, myosin, and adhesion dynamics changing during cell extrusion? How do cells get extruded, and yet the epithelial barrier is maintained? Currently, the authors jump to conclusions without characterizing the process itself, which makes the following experiments, describing perturbations, rather preliminary.

2. Why are the results from Figures 1B and 2C drastically different? In figure 1B, the number of putative MCCs decreases to roughly 30% (from the original 250 cells/mm2 to approximately 75 cells/mm2) by stage 46 while in panel 2C, the percentage of GFP-positive embryos by stage 46 is close to 100%. Counting the number of embryos having at least one positive GFP cell does not seem like a statistically robust method. One could understand that the RFP to GFP conversion is not 100% efficient, but assuming that the efficiency is constant between stages, the authors should quantify the number of GFP-positive cells. Currently the method presents itself as not very sensitive and biased: an embryo with just a few MCCs is still scored as GFP positive.

3. The concept of stable RFP remnants introduced by the authors is quite puzzling. First, it is unclear how authors quantify the apical and basal extrusions. Is this based on the presence of RFP remnants, or did authors live-imaged the process and score the basal and apical extrusions? The authors do not describe the formation of remnants, and thus it is speculative how the formation of remnants reflects the extrusion process. Second, how specific are these remnants, could authors exclude that other processes than extrusion trigger the formation of remnants? I'm unsure whether the number of RFP clusters per mm2 is a good enough proxy for basal extrusion. The number of debris resulting from each extrusion event should be highly variable, one should be very cautious when comparing different treatments. While the authors report that RFP debris is long-lived in the tissue and that these do not seem to be cleared from the tissue, the persistence (or lifetime) of debris in the tissue has not been documented in detail. Altogether while the number of debris can be used as a quick proxy, it is hard to see how the number of debris could be a clear indicator of cell extrusion that can be used to perform statistical analysis to compare different conditions.
I would argue that a different measure for the extent of basal extrusion should be found.

4. Third, what is happening with the MCC transdifferentiating to goblet cells as described in Tasca et al 2021, are these cells also lost? This should also be debated in the discussion. Generally, it has not been clearly characterized how the remnants are formed and how one could use them to learn something about the process of extrusion. Currently, the description of MCCs' basal extrusion is too speculative.

5. Similarly, when talking about apical extrusion and anoikis: Here the authors should define why apical extrusion of MCCs is driven by anoikis: what are the markers that define this particular process of cell death? In Gudipaty et al (2017), as referenced by the authors, sphingosine-1-phosphate (S1P) is produced and secreted by the extruded cells to activate RhoA-mediated actin contractility which squeezes the cells out of the tissue. Do the extruding MCCs secrete S1P?
Also, it is unclear whether these cells are dead or alive when they get extruded.

© 2023. Published by The Company of Biologists under the terms of the Creative Commons Attribution License (https://creativecommons.org/licenses/by/4.0/).

6. Moreover, panels 3A and 3B are the centerpieces of the paper, as they describe the two different mechanisms by which MCCs get extruded. The quality of the images is, unfortunately, underwhelming. Including 3D renderings or using XZ (orthogonal) sections of the extruded MCC might be useful to show more clearly the different extrusion mechanisms. In figure 3B, a contracting apical actin ring is maintained after the MCC is apically extruded. What is occupying that space? Does the epithelium simply leave a gap that is later resolved?

7. While the part about animal caps is interesting as it shows that MCC identity can be maintained ex vivo and in the absence of mesodermal signals for significantly longer periods of time, these results are not new as they just reproduce the main findings of Tasca et al paper, where lateral line ablation sustains MCC identity. These results should be moved to a supplementary figure.

8. While the transplantation assays are interesting, and the technical prowess of the authors should be admired, I'm not sure that they really entail the interpretation proposed by the authors. What is happening here with the signals from the lateral line?

9. The observation that the MCC transcriptional program is protective of MCC loss is exciting! However, the authors haven't shown that it is indeed the case. While the authors present data suggesting that blocking transcription stops the deciliation-induced rebooting (Supp Figure 1), it would be nice to see that MCC identity is actually sustained.

10. Piezo 1 is involved in regulating MCC apical area and centriole number (Kulkarni et al 2021) – how are the results of this paper compatible with this? This should be included in the discussion.

11. What is the evidence for cell crowding? The authors propose that cell crowding triggers Piezo1 activation to induce apical extrusion, but the authors provide no experimental evidence of cell crowding. This is very important because it would help define what is the physiological (presumably growth and cell crowding) actor driving extrusion. More experiments are needed to dissect the contributions of the Notch basal and Piezo1 apical extrusion mechanisms in MCC removal. If there is a differential temporal contribution of these mechanisms then adding inhibitor and washing out experiments could help define this: e.g. one possible experiment is to add the Notch inhibitor till stage 46, wash out and then add the Piezo1 inhibitor.

12. In the discussion, the authors point to the existence of selective pressure. While this argument is somewhat compelling, it would be more convincing if the authors could comment if the loss of MCC observed in other amphibian embryos. If this is the case, then there would be some evidence to support the "strong selective pressure" argument.

Additional comments and minor comments:
1. The presentation of the paper is rather on the low side:
A) Figure quality is, unfortunately, poor
B) Please consider changing the font type for describing figures
C) Please change the color scheme red/green to a colorblind-friendly color palette
D) The paper is full of typos. For example, Piezo 1 is frequently misspelled throughout the manuscript.
E) Placing the citations of the papers the authors are thinking about here would be highly informative for the readers. For example: "In most systems, cell extrusion is normally unidirectional and only occurs bidirectionally in disease or experimental conditions."
F) Information detailing the image analysis (such as quantification of the extrusion events) should be included in the methods section.
G) While the authors include the number of cells and embryos used in each quantification, they forgot to mention the number of experiments performed.
H) Supplementary movies are missing time stamping and scale bars.
I) Missing scale bars in close-up images J) Figure 3: The authors shouldn't plot the ratios for each embryo. They should present the data for all the different embryos by pooling all the events together from the different experiments. If they choose to do so, they could also show individual data for each embryo in the supplementary data.
K) Figure 1A, pix/sec. Pixel size has to be determined, and the unit change accordingly.

© 2023. Published by The Company of Biologists under the terms of the Creative Commons Attribution License (https://creativecommons.org/licenses/by/4.0/).

L) Movie S1: The drift in the movie makes it hard to understand what the neighboring cells are doing during basal extrusion.
M) Movie S2: The drift in the movie makes it hard to understand what the neighboring cells are doing during apical extrusion. Also the volumetric analysis could also help describe apical extrusion better - it looks like there's an actin ring being formed around the apically extruding cell, but the diameter of the ring seems to be smaller than the diameter of the cell N)
Movie S3: Include stills from movie S3 as a supplementary figure.
O) There is no information about statistics on figures and within material and methods. For example, in Figure 2C, does it represent mean and SD/SE? The "SD/SE" at stage 48 is missing the bottom bar.
P) The a-tub mem-RFP transgenic line has not been described in the referenced paper. Is it actually a membrane transgene? Should it label axonemes in that case?

Reviewer 2

Advance summary and potential significance to field

The effective removal of unwanted cells via extrusion while sustaining a functional barrier is essential for remodeling and maintenance of epithelial tissues. Cell extrusion is thought to typically occur in one direction, while bidirectional extrusion is associated with pathological states such as cancer. Here Ventrella et al. describe a mechanism by which multi-ciliated cells (MCCs) are eliminated from the epidermis of Xenopus embryos by distinct mechanisms at different times in development. The authors demonstrate that the MCCs are utilized for fluid flow early on, and then are progressively lost by extrusion as development proceeds. Intriguingly, they show the direction in which the MCCs extrude from the tissue changes from going basally in early development to apically later in development. To test if the directionality of MCC extrusion uses distinct molecular mechanisms, they use both overexpression of Notch-intracellular domain in the MCCs and skin transplantation experiments to show that MCCs display a cell-intrinsic age dependent receptivity to Notch that promotes extrusion in the basal direction early in development. Further, experiments to promote or restart the MCC transcriptional program suggest it is protective against MCC cell loss by extrusion. Lastly, the authors show that MCCs switch to being eliminated via apical extrusion that is under the control of the mechnosensitive ion channel Piezo 1. The authors' conclusions are largely supported by the data and the experiments are thoroughly controlled throughout. Taken together, these results provide new insights into the regulation of cell elimination by extrusion and highlight a role for this process in epithelial tissue remodeling during development.

Comments for the author

Points for Clarification:
1) The authors mention that they "can observe the remnants of MCCs that have basally delaminated and undergone apoptosis". Likewise, they state later in the paper "a live apical extrusion driven by a Piezo1 mechanosensory mechanism". Yet markers of caspase activity or apoptosis are not shown to support these claims. Thus, it would be important for the authors to demonstrate the activation of caspase-3 or progression through apoptosis via tunel staining, or the lack thereof, to fully support this claim. Additionally, it would be helpful for the authors to discuss the hallmarks of apoptosis and their presence in MCCs to better define the mode of cell death of these cells during process of basal extrusion.
2) In Fig 3b, it appears there is a slight difference in the timescales at which basal (<20min) vs apical (>20min) extrusion occurs. At 20 min, the cell has already extruded in the basal direction and fragmented into extracellular vesicles or apoptotic bodies, while the cell going apically is still attached and progressing through the extrusion process. Can the authors comment if this difference in timing is consistently observed for the different modes of extrusion, or is due to the representative images provided? This information could be useful to further distinguish the mechanisms driving basal vs apical extrusion.
The authors may also wish to compare their timescale results to those of extrusion observed in other in vivo or culture systems in the discussion.
3) In Fig 4a-b, the authors use elegant skin transplant experiments to suggest a cell intrinsic age-based feature to the Notch signal. While I appreciate the technically demanding aspect of

© 2023. Published by The Company of Biologists under the terms of the Creative Commons Attribution License (https://creativecommons.org/licenses/by/4.0/).

these experiments, how can the authors exclude the possibility that the younger mesoderm was also transplanted, and therefore influencing Notch signaling in the tissue? Additionally, a mock transplant control using the same stage to rule out effects from the procedure itself or the converse, older tissue grafted onto younger embryos, would also help to provide additional support to this point.
4) After treatment with the Piezo1 agonist, the authors observe no significant difference in the number of apoptotic vesicles and use this a proxy to suggesting that basal extrusion still occurred normally. To determine the changes in the frequency or number of cells extruding in different directions, a direct quantification of the number of apical vs basally extruding cells would greatly aid the reader in assessing the change in directionality.
5) The authors focus largely on the intrinsic mechanisms of the extruding cells, but the neighboring cells are also known to play a key role in facilitating extrusion. Can the authors comment on the role of the neighboring cells epithelial cells in the removal of the MCCs via apical or basal extrusion. Are there distinct changes in the cytoskeleton in neighboring cells at different times in development that may be associated or contribute to either basal or apical extrusion?
6) While I agree that the data supports that MCCs must first abandon their MCC transcriptional program to undergo extrusion via the notch pathway, it seems like a bit of an overstatement to suggest that they are trans-differentiating. The provided data suggest the MCCs are returning to an early state to initiate a ciliogenesis program, as opposed to the MCCs turning into a different cell type (no data is provided to assess this). The authors should consider clarifying point.

Minor point:
In Fig 7a-b, can the authors comment on the overlap of Tub-MDCIDAS and Tub-memRFP as this would serve as an important control for specificity of the assay of targeted expression in MCCs.

Reviewer 3

Advance summary and potential significance to field

This is a clear and well documented analysis of the extrusion of cliated cells from the frog epidermis. The epidermis uses two modes, both apical and basal extrusion, and this manuscript argues persuasively that basal extrusion and disintegration of cells occurs due to Notch signaling. The manuscript uses a variety of transient transgenic (and one true transgenic) approaches, with immunofluorescence and other suitable techniques that lead to compelling data. The second mechanism uses piezo, and is analyzed by gain and loss of piezo function. Interestingly, the apical extrusion seems to leave a hole in the epidermis, but maybe the reporter is sub-cortical. Both mechanisms are well documented, with the grafting experiment also demonstrating that youthful epidermis is more resistant than older epidermis to basal extrusion signals.

Given the importance and widespread distribution of the ciliated epidermis, and interest in this level of regulation, the demonstration that there are difference mechanisms for basal and apical extrusion work should be of interest to a wide audience. The movies and beautiful documentation will draw people in, and once the many typos are fixed the manuscript is very clearly written, though often verbose. Initially I thought that this may have gone through the wringer at another journal, because it is publication ready but the typos suggests that the manuscript may not even have been read by all the authors. (peizo nuceli, mechansms, figgure, suffecient, decommiting and suggests (subject verb agreement))

But overall, I am enthusiastic about publication of the work in Development.

Comments for the author

Since the journal demands that I put something in this box, I will repeat the request for copy-editing, or running through spellcheck!

© 2023. Published by The Company of Biologists under the terms of the Creative Commons Attribution License (https://creativecommons.org/licenses/by/4.0/).

First revision

Author response to reviewers' comments

We appreciate the reviewers' positive comments about our work as well as their suggestions for improvement. We have made considerable changes to the figures and text that we believe has significantly improved the manuscript. However, we believe that some of the insightful comments would best be achieved by detailed analysis and new experimental directions that are beyond the scope of the current manuscript. Below is a point-by-point response to the reviewer comments.

Reviewer 1

1. The authors should describe in further detail the cellular mechanisms that govern bidirectional cell extrusion. This is the focus of the paper, yet largely not addressed by the authors. Many important questions arise when thinking about the cell extrusion process. Is it cell autonomous, or there is the contribution of the neighboring cells? How are actin, myosin, and adhesion dynamics changing during cell extrusion? How do cells get extruded, and yet the epithelial barrier is maintained? Currently, the authors jump to conclusions without characterizing the process itself, which makes the following experiments, describing perturbations, rather preliminary.

We fully agree with the reviewer that there are a lot of important details that have yet to be addressed regarding the cell biology of these cell extrusion events. However, we believe a proper detailed analysis of myosin, adhesion dynamics and neighbor contributions are beyond the scope of the current manuscript. We hope to address all of those points in future work.

2. Why are the results from Figures 1B and 2C drastically different? In figure 1B, the number of putative MCCs decreases to roughly 30% (from the original 250 cells/mm2 to approximately 75 cells/mm2) by stage 46 while in panel 2C, the percentage of GFP-positive embryos by stage 46 is close to 100%. Counting the number of embryos having at least one positive GFP cell does not seem like a statistically robust method. One could understand that the RFP to GFP conversion is not 100% efficient, but assuming that the efficiency is constant between stages, the authors should quantify the number of GFP-positive cells. Currently, the method presents itself as not very sensitive and biased: an embryo with just a few MCCs is still scored as GFP positive.

These two graphs quantify very different things. 1B is quantifying the total number of MCCs using either acetylated tubulin or tgTub-memRFP. The generation of the transgenic labeling in Figure 2C is mosaic. Therefore, it does not make sense to quantify total MCC number as some embryos have less transgenically labeled cells than others from the start. We instead wanted to use the lowest possible bar of quantifying embryos that have ANY GFP positive cells. What we report is that in over 100 embryos by ST48 we essentially find none with ANY GFP positive cells indicating the complete loss of MCCs. The main point of this figure is that if these cells were transdifferentiating they would still be present, which they are not. We have added the following text to explain these data more thoroughly. “Although this analysis may not provide an accurate reflection of the total number of MCCs, we regard it as a conservative benchmark for identifying any cells maintained after trans- or de-differentiation.” We have also added more detail to the experimental description.

3. The concept of stable RFP remnants introduced by the authors is quite puzzling. First, it is unclear how authors quantify the apical and basal extrusions. Is this based on the presence of RFP remnants, or did authors live- imaged the process and score the basal and apical extrusions? The authors do not describe the formation of remnants, and thus it is speculative how the formation of remnants reflects the extrusion process. Second, how specific are these remnants, could authors exclude that other processes than extrusion trigger the formation of remnants? I'm unsure whether the number of RFP clusters per mm2 is a good enough proxy for basal extrusion. The number of debris resulting from each extrusion event should be highly variable, one should be very cautious when comparing different treatments. While the authors report that RFP debris is long-lived in the tissue and that these do not seem to be cleared from the tissue, the persistence (or lifetime) of debris in the tissue has not been documented in detail. Altogether while the number

© 2023. Published by The Company of Biologists under the terms of the Creative Commons Attribution License (https://creativecommons.org/licenses/by/4.0/).

of debris can be used as a quick proxy, it is hard to see how the number of debris could be a clear indicator of cell extrusion that can be used to perform statistical analysis to compare different conditions. I would argue that a different measure for the extent of basal extrusion should be found.

We have added more experimental detail to how these events were quantified. For the purposes of the original Figure 3D, the data was quantified from live light sheet movies using low resolution objectives. While not beautiful, we can distinguish between a cell that extrudes apically and disappears versus basally generating the RFP cluster. An example was provided in Movie S3 (now Movie S4). To address the reviewers concerns regarding the lack of documentation of the longevity of the RFP+ clusters we have now provided an additional Supplemental Figure that tracks several clusters over extended periods of time (13.3, 16.6 and 18.3 Hrs) until the cell goes out of frame due to drift (Supplemental Figure 2A-C). Additionally, we added a more extensive movie from S2C (Supplemental Movie 2). This new documentation supports the validity of our previous quantifications on fixed samples. While it is certainly possible that two neighboring MCCs that basally extrude around the same time could potentially be quantified as a single cluster, the overall consistent spacing of the MCCs across the epithelium, coupled with the large number of MCCs and embryos that we quantified would argue that any hypothetical error would not statistically alter our results.

4. Third, what is happening with the MCC transdifferentiating to goblet cells as described in Tasca et al 2021, are these cells also lost? This should also be debated in the discussion. Generally, it has not been clearly characterized how the remnants are formed and how one could use them to learn something about the process of extrusion. Currently, the description of MCCs' basal extrusion is too speculative.

The MCC specific expression of RFP coupled with the lack of RFP+ clusters early in development (prior to extrusion) and the spacing of RFP+ clusters matching the spacing of MCCs all argue that these clusters are MCC remnants. We have added a new side projection movie that shows the basal extrusion and RFP+ cluster formation more clearly (Movie S1). Additionally, our newly added supplemental data (Figure S2 and Movie S2, as well as Movie S4) provide a better description of basal extrusion. We agree with the reviewer and have added a more in-depth discussion of our data in relation to the Tasca paper.

Similarly, when talking about apical extrusion and anoikis: Here the authors should define why apical extrusion of MCCs is driven by anoikis: what are the markers that define this particular process of cell death? In Gudipaty et al (2017), as referenced by the authors, sphingosine-1-phosphate (S1P) is produced and secreted by the extruded cells to activate RhoA-mediated actin contractility, which squeezes the cells out of the tissue. Do the extruding MCCs secrete S1P? Also, it is unclear whether these cells are dead or alive when they get extruded.

While we agree that the characterization of S1P and RhoA are important directions, we feel that a proper analysis of these pathways is beyond the scope of the current work. We hope to address these issues in more detail in future work. Regarding whether the cells are alive or dead we have added new supplemental data showing that the RFP + clusters can stain positive for cleaved caspase 3. However, our analysis of apoptotic cells is performed on fixed samples which precludes us from identifying cells as apically extruding which is based on live imaging. Our assertion that the cells are “live” is based on the observation that we have seen beating cilia up to the point of extrusion (e.g Movie S3). Furthermore, when we block Piezo1 we only see a slight increase in basal extrusion, and most of the cells are maintained and remain alive. This does not preclude the possibility that Piezo1 drives a distinct apoptosis but it does suggest that the majority of these cells are regulated differently from the basal apoptotic pool. We have added text to the discussion to acknowledge this possibility.

6. Moreover, panels 3A and 3B are the centerpieces of the paper, as they describe the two different mechanisms by which MCCs get extruded. The quality of the images is, unfortunately, underwhelming. Including 3D renderings or using XZ (orthogonal) sections of the extruded MCC might be useful to show more clearly the different extrusion mechanisms. In figure 3B, a contracting apical actin ring is maintained after the MCC is apically extruded. What is occupying that space? Does the epithelium simply leave a gap that is later resolved?

© 2023. Published by The Company of Biologists under the terms of the Creative Commons Attribution License (https://creativecommons.org/licenses/by/4.0/).

The quality of the images is indeed not as good as we typically publish. However, this is not due to the microscopes or the preparation, but the biological question being addressed. Any given cell extrudes sometime over a several day period, which means finding a cell extruding via high spatial and temporal resolution confocal microscopy is extremely challenging. For this reason we have put considerable effort into developing light sheet paradigms which are great for observing and quantifying events but lack the aesthetics of confocal. Additionally, injection of fluorescent probes via standard Xenopus methods of DNA or mRNA injection begin to fade by these late stages. The images provided are the best we were able to get but certainly lack the temporal and spatial resolution that we would normally be able to achieve. However, we have remade the movie to contain both apical and side projections simultaneously for clarification.

7. While the part about animal caps is interesting as it shows that MCC identity can be maintained ex vivo and in the absence of mesodermal signals for significantly longer periods of time, these results are not new as they just reproduce the main findings of Tasca et al paper, where lateral line ablation sustains MCC identity. These results should be moved to a supplementary figure.

While the Tasca paper indeed suggested that the Notch signal was stemming from the underlying mesoderm, we felt that this added to that finding by showing the loss of the mesoderm in fact leads to a maintenance of MCCs (which others have shown), but which is subsequently lost with the addition of activated Notch. We agree that this is not central to the paper and could be included in supplemental and have accordingly moved it there (new Figure S3).

8. While the transplantation assays are interesting, and the technical prowess of the authors should be admired, I'm not sure that they really entail the interpretation proposed by the authors. What is happening here with the signals from the lateral line?

There is variation on where the explants are positioned relative to the host embryo so it is hard to unequivocally state where all the transplants are relative to the lateral line. Importantly, however, our quantifications are performed at ST48 which is well beyond the ST when some MCCs are lost via lateral line signaling so any quantifications we have made take into account the potential loss from the lateral line.

9. The observation that the MCC transcriptional program is protective of MCC loss is exciting! However, the authors haven't shown that it is indeed the case. While the authors present data suggesting that blocking transcription stops the deciliation-induced rebooting (Supp Figure 1), it would be nice to see that MCC identity is actually sustained.

The maintenance or loss of MCC fate is likely a spectrum which would make defining a meaningful cutoff challenging in this scenario. The best way to address this concern would be to perform a detailed scRNA seq analysis of these tissues which is unfortunately beyond the scope of the current project. However, the presence of cilia as well as TgTub-memRFP suggests that these cells are maintaining their fate. However, we have tempered our claim, to state that we "suggest" MCC transcriptional maintenance is protective.

10. Piezo 1 is involved in regulating MCC apical area and centriole number (Kulkarni et al 2021) – how are the results of this paper compatible with this? This should be included in the discussion.

Piezo 1 is a versatile protein that functions in a wide range of biological contexts. Given the vastly different stages that are being addressed here, I am not sure there are a lot of meaningful comparisons that can be drawn. That said, I agree that it is worth discussing and we have added text to the discussion that acknowledges these two phenotypes and places our data in the context of other functions for Piezo 1.

11. What is the evidence for cell crowding? The authors propose that cell crowding triggers Piezo1 activation to induce apical extrusion, but the authors provide no experimental evidence of cell crowding. This is very important because it would help define what is the physiological (presumably growth and cell crowding) actor driving extrusion. More experiments are needed to dissect the contributions of the Notch basal and Piezo1 apical extrusion mechanisms in MCC removal. If there is a differential temporal contribution of these mechanisms, then adding inhibitor and washing out

© 2023. Published by The Company of Biologists under the terms of the Creative Commons Attribution License (https://creativecommons.org/licenses/by/4.0/).

experiments could help define this: e.g. one possible experiment is to add the Notch inhibitor till stage 46, wash out and then add the Piezo1 inhibitor.

The suggestion of treating embryos until ST 46 with the Notch inhibitor, followed by the Piezo inhibitor was an interesting suggestion and one that we were eager to perform. Unfortunately, despite numerous attempts the embryos have all died. One interpretation of this result is that the loss of MCCs is important for overall epithelial integrity and that the inhibition of either pathway can be tolerated but that blocking both results in a loss of integrity and ultimately death from overcrowding. However, due to the negative nature of this result and the speculative nature of this interpretation we have not included it. Furthermore, the Tasca et al paper quantified a trend towards higher cell counts / area as development continues and we have added this as a reference.

12. In the discussion, the authors point to the existence of selective pressure. While this argument is somewhat compelling, it would be more convincing if the authors could comment if the loss of MCC observed in other amphibian embryos. If this is the case, then there would be some evidence to support the “strong selective pressure” argument.

We have added text and references that indicate the evolutionary conserved nature of MCC loss in amphibians.

Additional comments and minor comments:

1.The presentation of the paper is rather on the low side:
A) Figure quality is, unfortunately, poor
The timing of these events makes high resolution imaging challenging.

B) Please consider changing the font type for describing figures
We have remade all of the figures and have changed the font to Ariel.

C) Please change the color scheme red/green to a colorblind-friendly color palette
We have remade the figures to include more a color blind friendly palette. Additionally, we have added individual monochromatic images of relevant channels.

D) The paper is full of typos. For example, Piezo 1 is frequently misspelled throughout the manuscript.
We have performed more rigorous editing and hopefully fixed the many mistakes.

E) Placing the citations of the papers the authors are thinking about here would be highly informative for the readers. For example: “In most systems, cell extrusion is normally unidirectional and only occurs bidirectionally in disease or experimental conditions.”
We have added references.

F) Information detailing the image analysis (such as quantification of the extrusion events) should be included in the methods section.
We have added detail to the methods.

G) While the authors include the number of cells and embryos used in each quantification, they forgot to mention the number of experiments performed.
This has now been added to the legends.

H) Supplementary movies are missing time stamping and scale bars.
We have added time stamps and scale bars.

I) Missing scale bars in close-up images
We feel this is redundant and given how tight the space is in the inlays we have left them out.

J) Figure 3: The authors shouldn’t plot the ratios for each embryo. They should present the data for all the different embryos by pooling all the events together from the different experiments. If they choose to do so, they could also show individual data for each embryo in the supplementary data.

© 2023. Published by The Company of Biologists under the terms of the Creative Commons Attribution License (https://creativecommons.org/licenses/by/4.0/).

We have now re-analyzed the live light sheet imaging data to quantify the rate of extrusion (i.e. the number of events / mm^2/ hr) (New Figure 3D). We have also included a graph of the percent of basal events (Figure 3E), which is similar to before but which we think is clearer.

K) Figure 1A, pix/sec. Pixel size has to be determined, and the unit change accordingly.
We have converted the data to µm / sec

L) Movie S1: The drift in the movie makes it hard to understand what the neighboring cells are doing during basal extrusion.
We realigned this movie.

M) Movie S2: The drift in the movie makes it hard to understand what the neighboring cells are doing during apical extrusion. Also the volumetric analysis could also help describe apical extrusion better - it looks like there's an actin ring being formed around the apically extruding cell, but the diameter of the ring seems to be smaller than the diameter of the cell
We realigned this movie and provided both apical and side projections simultaneously.

N) Movie S3: Include stills from movie S3 as a supplementary figure.
We have added stills to Supplemental Figure S2D.

O) There is no information about statistics on figures and within material and methods. For example, in Figure 2C, does it represent mean and SD/SE? The"SD/SE" at stage 48 is missing the bottom bar.
We have added statistical information where missing.

P)The a-tub mem-RFP transgenic line has not been described in the referenced paper. Is it actually a membrane transgene? Should it label axonemes in that case?
We apologize for the oversight. This construct is available form the NXR and we have now properly attributed it. It does label the ciliary membrane as can be seen in the side projections from 3A and B. It is hard to see in apical projections as the cell membrane staining is stronger than the cilia staining and thus obscures it. In live imaging it can be hard to see due to the speed of cilia beating and in the low-resolution light sheet imaging cilia are not typically discernable.

Reviewer 2 Comments for the Author: Points for Clarification:
1) The authors mention that they "can observe the remnants of MCCs that have basally delaminated and undergone apoptosis". Likewise, they state later in the paper "a live apical extrusion driven by a Piezo1 mechanosensory mechanism". Yet, markers of caspase activity or apoptosis are not shown to support these claims. Thus, it would be important for the authors to demonstrate the activation of caspase-3 or progression through apoptosis via tunel staining, or the lack thereof, to fully support this claim. Additionally, it would be helpful for the authors to discuss the hallmarks of apoptosis and their presence in MCCs to better define the mode of cell death of these cells during process of basal extrusion.

We have added a supplemental figure showing RFP positive remnants that are positive for antibody staining of Cleaved Caspase 3. However, MCCs are not the only cells that are potentially undergoing apoptosis and we sometimes see Caspase 3 positive clusters that are not RFP positive, which we interpret as non-MCC apoptotic cells. Additionally, Cleaved Caspase 3 does not stain all phases of apoptosis so we also see RFP positive clusters that are not staining positive for Cleave Caspase 3. Finally, we have not observed Cleaved Caspase 3 staining in apically extruding cells although this would be challenging using our current experimental set up as we have not had success with live staining of apoptotic markers. While our added data indicates that the RFP positive clusters can represent apoptotic remnants, it also highlights the challenges of strictly using apoptotic markers for this analysis and supports our use of RFP positive clusters for quantification of MCC death. We have also added text to temper our claim as well as better describe our rationale. In particular, the newly added side projections of Movie S3 shows that the apically extruding MCC maintains motile cilia up until it extrudes.

2) In Fig 3b, it appears there is a slight difference in the timescales at which basal (<20min) vs apical (>20min) extrusion occurs. At 20 min, the cell has already extruded in the basal direction and

© 2023. Published by The Company of Biologists under the terms of the Creative Commons Attribution License (https://creativecommons.org/licenses/by/4.0/).

fragmented into extracellular vesicles or apoptotic bodies, while the cell going apically is still attached and progressing through the extrusion process. Can the authors comment if this difference in timing is consistently observed for the different modes of extrusion, or is due to the representative images provided? This information could be useful to further distinguish the mechanisms driving basal vs apical extrusion. The authors may also wish to compare their timescale results to those of extrusion observed in other in vivo or culture systems in the discussion.

In both apical and basal extrusion, the actual extrusion step is quite rapid. The discrepancy in time points in the two images (3A and 3B) was to highlight the fact that RFP positive clusters remain for some time. This point has now been made more rigorously with additional supplemental data (Supp Fig 2 and Movie S2). Additionally, we have added quantification (from light sheet imaging) that shows the rate of apical versus basal extrusion as a factor of time and area (Fig 3 D).

3) In Fig 4a-b, the authors use elegant skin transplant experiments to suggest a cell intrinsic age-based feature to the Notch signal. While I appreciate the technically demanding aspect of these experiments, how can the authors exclude the possibility that the younger mesoderm was also transplanted, and therefore influencing Notch signaling in the tissue? Additionally, a mock transplant control using the same stage to rule out effects from the procedure itself or the converse, older tissue grafted onto younger embryos, would also help to provide additional support to this point.

As noted, these experiments are quite challenging. We have put considerable effort into the proposed experiments but unfortunately, they are not currently feasible. At younger stages it is quite easy to separate the cell layers so that they remain intact and therefore I don't think there is much chance of mesoderm contamination from the younger tissue. However, the removal of the skin at ST28 is more challenging and while we can still decern a single layer, the removed skin immediately rolls up onto itself. This makes reusing this older skin untenable. While we can replace the removed older skin with younger skin that remains flat upon removal, we cannot do the reverse, nor can we do the mock transplant of older tissue onto older tissue. While we could do the mock of younger tissue onto younger tissue, our previous use of this model would argue that this tissue becomes well integrated and that this would not ultimately be an informative control. Given the multiple lines of evidence that support our claims we feel that our transplant studies as they stand still provide useful information despite the lack of these additional controls.

4) After treatment with the Piezo1 agonist, the authors observe no significant difference in the number of apoptotic vesicles and use this a proxy to suggesting that basal extrusion still occurred normally. To determine the changes in the frequency or number of cells extruding in different directions, a direct quantification of the number of apical vs basally extruding cells would greatly aid the reader in assessing the change in directionality.

Unfortunately, these experiments would be prohibitively expensive. Our live light sheet method of quantification is quite challenging as we cannot restrict the motion of the embryo. The use of either agarose or even our standard mounting procedure of squeezing the embryo between pieces of cover glass to restrict motion could potentially alter the ability of cells to extrude (e.g. if pushed against the glass there would be a barrier to extrusion). We therefore created a barrier to forward motion but otherwise left the embryo free. This required numerous attempts to capture embryos that didn't move outside the field of view. While not very efficient, the cost of doing many iterations of these movies on wild type embryos is insignificant. However, to repeat this approach in the presence of the Piezo1 agonist would be cost prohibitive.

5) The authors focus largely on the intrinsic mechanisms of the extruding cells, but the neighboring cells are also known to play a key role in facilitating extrusion. Can the authors comment on the role of the neighboring cells epithelial cells in the removal of the MCCs via apical or basal extrusion. Are there distinct changes in the cytoskeleton in neighboring cells at different times in development that may be associated or contribute to either basal or apical extrusion?

Given the literature on cell extrusion in other systems, it is certain that there is an important contribution from neighboring cells, which is observable in the actin ring from our example of apical extrusion Fig. 3B. While we agree that this is an important line of inquiry, we believe that a proper analysis is beyond the scope of the current paper. However, it is an important point and one

© 2023. Published by The Company of Biologists under the terms of the Creative Commons Attribution License (https://creativecommons.org/licenses/by/4.0/).

that we have add to the discussion as a current limitation and future goal.

6) While I agree that the data supports that MCCs must first abandon their MCC transcriptional program to undergo extrusion via the notch pathway, it seems like a bit of an overstatement to suggest that they are trans-differentiating. The provided data suggest the MCCs are returning to an early state to initiate a ciliogenesis program, as opposed to the MCCs turning into a different cell type (no data is provided to assess this). The authors should consider clarifying point.

We have added considerable text to the discussion in an attempt to clarify this point.

Minor point:
In Fig 7a-b, can the authors comment on the overlap of Tub-MDCIDAS and Tub-memRFP, as this would serve as an important control for specificity of the assay of targeted expression in MCCs.

We have remade this figure (now 6A-B) to include separate monochromatic images of both acetylated tubulin and Tub-memRFP that shows the extended expression of RFP in this context. However, these experiments were performed with an untagged version of MCIDAS so we do not have the exact requested overlap. However, our data in Figure S5 shows that mosaic expression of MCIDAS leads to mosaic expression of Tub-memRFP and when coupled with previously published work that shows tubulin mRNA expression is downstream of MCIDAS (Stubbs et al 2012) argues that the MCIDAS is driving the Tub- memRFP as claimed. We have described this in more detail and added this reference.

Second decision letter

MS ID#: DEVELOP/2023/201612

MS TITLE: Bidirectional multiciliated cell extrusion is controlled by Notch driven basal extrusion and Piezo 1 driven apical extrusion.

AUTHORS: Rosa Ventrella, Sun K Kim, Jennifer Sheridan, Aline C Grata, Enzo Bresteau, Osama A Hassan, Evelyn E Suva, Peter Walentek, and Brian Mitchell
ARTICLE TYPE: Research Article

I am happy to tell you that your manuscript has been accepted for publication in Development, pending our standard ethics checks.

Reviewer 1

Advance summary and potential significance to field

The revised manuscript addressed all the issues from the first submission.

Comments for the author

The revised manuscript addressed all the issues from the first submission.

Reviewer 2

Advance summary and potential significance to field

The effective removal of unwanted cells via extrusion while sustaining a functional barrier is essential for remodeling and maintenance of epithelial tissues. Cell extrusion is thought to typically

© 2023. Published by The Company of Biologists under the terms of the Creative Commons Attribution License (https://creativecommons.org/licenses/by/4.0/).

occur in one direction, while bidirectional extrusion is associated with pathological states such as cancer. Here Ventrella et al. describe a mechanism by which multi-ciliated cells (MCCs) are eliminated from the epidermis of Xenopus embryos by distinct mechanisms at different times in development. The authors demonstrate that the MCCs are utilized for fluid flow early on, and then are progressively lost by extrusion as development proceeds. Intriguingly, they show the direction in which the MCCs extrude from the tissue changes from going basally in early development to apically later in development. To test if the directionality of MCC extrusion uses distinct molecular mechanisms, they use both overexpression of Notch-intracellular domain in the MCCs and skin transplantation experiments to show that MCCs display a cell-intrinsic age dependent receptivity to Notch that promotes extrusion in the basal direction early in development. Further, experiments to promote or restart the MCC transcriptional program suggest it is protective against MCC cell loss by extrusion. Lastly, the authors show that MCCs switch to being eliminated via apical extrusion that is under the control of the mechnosensitive ion channel Piezo 1. Taken together, these results provide new insights into the regulation of cell elimination by extrusion and highlight a role for this process in epithelial tissue remodeling during development.

Comments for the author

The revised version of the manuscript “Bidirectional multiciliated cell extrusion is controlled by Notch driven basal extrusion and Piezo1 driven apical extrusion” by Ventrella et al. is improved and the authors have largely addressed the reviewers' critiques. Importantly, a more detailed visualization and quantification of apical versus basal extrusion of the MCCs is provided via additional supplemental movies and modifications to Fig 3. Given the focus on extrusion as the mechanism to eliminate the MCCs, these data should be described in a bit more detail. For instance, the rationale and use of LifeACT-GFP to visualize F-actin during extrusion is not mentioned in the results section. This provides the authors with an opportunity to highlight some of the hallmarks of extrusion in these data to support their conclusions, such as the presence of cellular rosettes and the multi-cellular ring of actin, that are not currently described. It could also be used to orient the reader with the positioning of the actin ring below or above the cell as a defining feature that promotes apical or basal extrusion, respectively. In my opinion, once this point has been addressed the article would be suitable for publication in Development.

© 2023. Published by The Company of Biologists under the terms of the Creative Commons Attribution License (https://creativecommons.org/licenses/by/4.0/).
